# Novel anaerobic selenium oxyanion reducers native to FGD wastewater for enhanced selenium removal

**DOI:** 10.1128/aem.01222-24

**Published:** 2025-03-14

**Authors:** Preom Sarkar, Meghan Beebe, Gita Bhandari, Jonas Wielinski, Gregory V. Lowry, Djuna Gulliver

**Affiliations:** 1 National Energy Technology Laboratory 17213 , Pittsburgh, Pennsylvania, USA; 2 NETL Support Contractor, Leidos, Pittsburgh, Pennsylvania, USA; 3 ORISE Fellow, Oak Ridge Institute for Science and Education 17215 , Oak Ridge, Tennessee, USA; 4 Carnegie Mellon University 6612 , Pittsburgh, Pennsylvania, USA; Washington University in St. Louis, St. Louis, Missouri, USA

**Keywords:** FGD, wastewater, anaerobic, selenium reduction, selenium nanoparticle, metagenome, taxonomy, biogenic selenium

## Abstract

Biological treatment is a recognized approach for removing selenate and selenite oxyanions present in flue gas desulfurization (FGD) wastewater. However, the knowledge of the specific microbial species or communities responsible for reducing water-soluble selenium oxyanions to insoluble elemental selenium remains limited. In addition, the selenium oxyanion reduction genes and pathways have yet to be understood in these wastewaters. This study characterizes selenium oxyanion-reducing bacteria (SeRB) native to FGD wastewater, and the resulting elemental selenium particles formed. By selecting native SeRB microbes in a defined media, a novel resolution of these organisms has been achieved. This research identifies previously unrecognized selenium oxyanion-reducing capabilities in *Anaerosolibacter*, alongside predominant SeRB from *Mesobacillus* and *Tepidibacillus* genera. This work encompasses both 16S and metagenomic techniques to recover novel metagenome-assembled genomes, distinct to this environment. The biogenic selenium produced by these organisms was predominantly of elemental selenium, either amorphous or with a hexagonal structure. This study identifies the SeRB present in FGD wastewater and characterizes their selenium products, offering crucial insights to enhance the efficiency of biological treatment strategies and the potential of selenium recovery from this industrial waste.

**IMPORTANCE:**

This is the first report on the culturability and recovery of taxonomic and metabolic information of the anaerobic selenium oxyanion-reducing bacteria (SeRB) in flue gas desulfurization (FGD) wastewater. Selenium is a regulated contaminant in FGD wastewater found on average to be 3,130 µg/L that must be removed to meet EPA discharge limits of 16 µg/L (D. B. Gingerich, E. Grol, and M. S. Mauter, Environ Sci Water Res Technol 4:909–925, 2018, https://doi.org/10.1039/C8EW00264A; also see U.S. EPA EPA-821-R-20-001, 2020). Better understanding of anaerobic SeRB and the microbial community in FGD wastewater is needed to harness their full potential for the bioremediation and recovery of selenium from FGD wastewater. Optimizing the biotreatment strategies for these wastewaters promises to yield cleaner and healthier waterways and ecosystems, even as the United States undergoes a shift in its energy landscape.

## INTRODUCTION

Coal accounts for ~20% of the United States’ (U.S.) electricity generation (1). Coal-fired power plants employ flue gas desulfurization (FGD) technologies to mitigate the release of sulfur oxide and nitrous oxide gasses resulting from coal combustion. While FGD systems play a crucial role in routine plant operation, the wastewater they generate contains additional contaminants that the Environmental Protection Agency (EPA) regulates through the Effluent Limitation Guideline (ELG) rules, imposing strict restrictions on discharges of various toxic compounds such as mercury and arsenic.

Of particular concern is selenium, a common element in coal, known for its capacity to induce harm at low concentration. In FGD effluent, concentrations of selenium range from 200 to 7,000 µg/L, far surpassing ELG discharge limits of 16 µg/L (2, 3). Cost-effective methods to remove selenium from these wastewaters prior to discharge are crucial to allow CFPPs to remain operational while maintaining US standards.

Selenium is a nonmetal chemical element with atomic number 34 with oxidation states ranging from −II to +VI. Selenium exists in various inorganic and organic species with distinct physicochemical properties that are influenced by environmental redox conditions (4). The predominant selenium species in FGD wastewater are selenate (SeO_4_^2−^) and selenite (SeO_3_^2−^) (5). These species are the most water-soluble selenium species and are considered to be the most toxic inorganic species of selenium due to their high bioavailability (6). However, in a pure elemental form (Se^0^), selenium is a valuable resource, applied in sectors such as medicine, therapeutics, environmental remediation, and electronics (7). This research provides insights into the selenium in FGD wastewater that can be removed and recovered from this wastewater if the microbial community is characterized and leveraged properly.

Microbial reduction of selenium oxyanion species occurs through two distinct steps, commencing with the reduction of selenate (+VI) to selenite (+IV), followed by the reduction of selenite to elemental selenium. Microbial selenium oxyanion reduction has been very well studied and is thought to be ubiquitous in a variety of environments (8). Previous reports have demonstrated that some organisms, such as *Thauera selenatis* (sourced from agricultural drainage water) can reduce selenate, some organisms such as *Shewanella onedinsis MR-1* (sourced from lake sediment) can reduce selenite, and some organisms such as *Desulfurispirillum indicum* (sourced from river sediment) can reduce both selenate and selenite (9–11). There has yet to be an organism identified that can carry out a reaction such that selenate is transformed directly to Se^0^ without the production of the intermediate selenite (4).

The bacterial species responsible for selenium reduction have not been identified for FGD wastewater even though biological treatment is considered the best available technology to treat FGD wastewater (12). Researchers have previously described the aerobic microbial communities in this wastewater, but this is not indicative of the metabolic functions of anaerobic organisms in FGD wastewater that can be leveraged to treat this water through anaerobic reduction of the selenium oxyanion species present (13). This work seeks to provide a novel resolution of these such organisms in this type of wastewater (4, 13)

This study seeks to taxonomically classify the selenium oxyanion-reducing bacteria (SeRB) of FGD wastewater, understand more about their functional capabilities via metagenomic sequencing, and characterize their biogenic selenium products. Here, we biologically selected SeRB native to FGD wastewater by providing selenate or selenite as terminal electron acceptors and analyzed the metagenomes of the organisms promoted in this process. The resulting solids formed were characterized by scanning electron microscopy (SEM) with energy dispersive spectrometry (EDS), x-ray diffraction (XRD), and single-particle inductively coupled plasma time-of-flight mass spectrometry (spICP-TOFMS). From our amendment reactors, we were able to determine that there are distinct microbial communities that utilize selenate and microbial communities that use selenite. Understanding more about these organisms can lead to the engineering of biological treatment systems that better leverage these microbes to remove and remediate selenium from these wastewaters.

## RESULTS AND DISCUSSION

### FGD microbial community is taxonomically different than amendments

To understand the anaerobic microbial community, FGD wastewater was incubated at anaerobic conditions at 40°C for 7 days and then subjected to chemical and microbial analysis.

The pH was circumneutral at an average of 6.6 (±0.1) for the anaerobic FGD, respectively. The total dissolved solids (TDS) concentration was approximately 16,000 mg/L. Major anions consisted of chloride 221 mM and sulfate 33 mM. Major cations consisted of magnesium 106 mM, calcium 28 mM, and sodium 46 mM.

To assess microbial composition, 16S gene amplicon sequencing was utilized, yielding a range of 5,239–7,300 reads per sample. Figure S1 in the supplemental material depicts the taxonomy and relative abundance of the most prevalent organisms in anaerobic FGD sample. Results from 16S sequencing demonstrate that in the anaerobic FGD community, the dominant organisms in these samples were from the order Peptostreptococcales-Tissierellales, which represented ~55% of the community, and the genus *Acetobacterium*, which represented ~24% of the community. Peptostreptococcales-Tissierellales is part of the class Clostridia. Organisms that belong to the class of Clostridia are spore-forming anaerobic and aerotolerant bacilli (14). This suggests an ability to withstand the environmental changes through the FGD wastewater treatment train despite the variation in operating conditions and biological mitigation processes. The second most abundant organism in this community is from the genus *Acetobacterium*, an anaerobic bacterium that primarily produces acetic acid (15). Although further study is needed, this microorganism could potentially provide a carbon source for SeRBs in the FGD wastewater treatment system, which also happened to be the carbon source in this experiment.

The anaerobic FGD fluid contains low microbial loads with high alpha diversity. To determine the total microbial abundance, droplet digital PCR (ddPCR) was conducted on all samples. The average microbial load for the samples was found to be 7.4 × 10^1^ 16S rRNA gene copies/mL (Table 1). The anaerobic FGD community also had a high alpha diversity with a Chao1 index value of 46.3 and a Shannon richness of 2.9. The anaerobic FGD community is largely different than that of the selenium oxyanion amended reactors in this study, demonstrating a presence of selenium-reducing bacteria in this wastewater, but at a small relative abundance in the original community.

**TABLE 1:** Total ASVs, averaged alpha diversity, and averaged microbial abundance measurements^*a*^

Sample	Total ASVs	Chao1	Shannon entropy	16S copies/mL
Anaerobic FGD	65	46.3	2.9	7.4E+01
Selenate				
TR0	27	17.7	2.0	5.3E+06
TR1	10	6.3	1.3	7.1E+06
TR2	12	6.7	0.9	1.9E+07
TR3	17	8.3	1.5	4.0E+03
TR4	12	6.3	0.8	1.4E+04
Selenite				
TR0	26	12.7	1.2	5.6E+02
TR1	14	7.0	0.3	2.6E+07
TR2	17	8.7	0.5	1.3E+05
TR3	13	5.7	0.4	1.6E+07
TR4	2	1.3	0.0	1.9E+07

^*a*^ Metrics were averaged among the triplicate data. Alpha diversity metrics (Chao1 and Shannon entropy) were calculated after sequence libraries were resampled to a depth of 2,791 sequences.

### Distinct microbial communities arise for each selenium oxyanion

**Fig 1:**
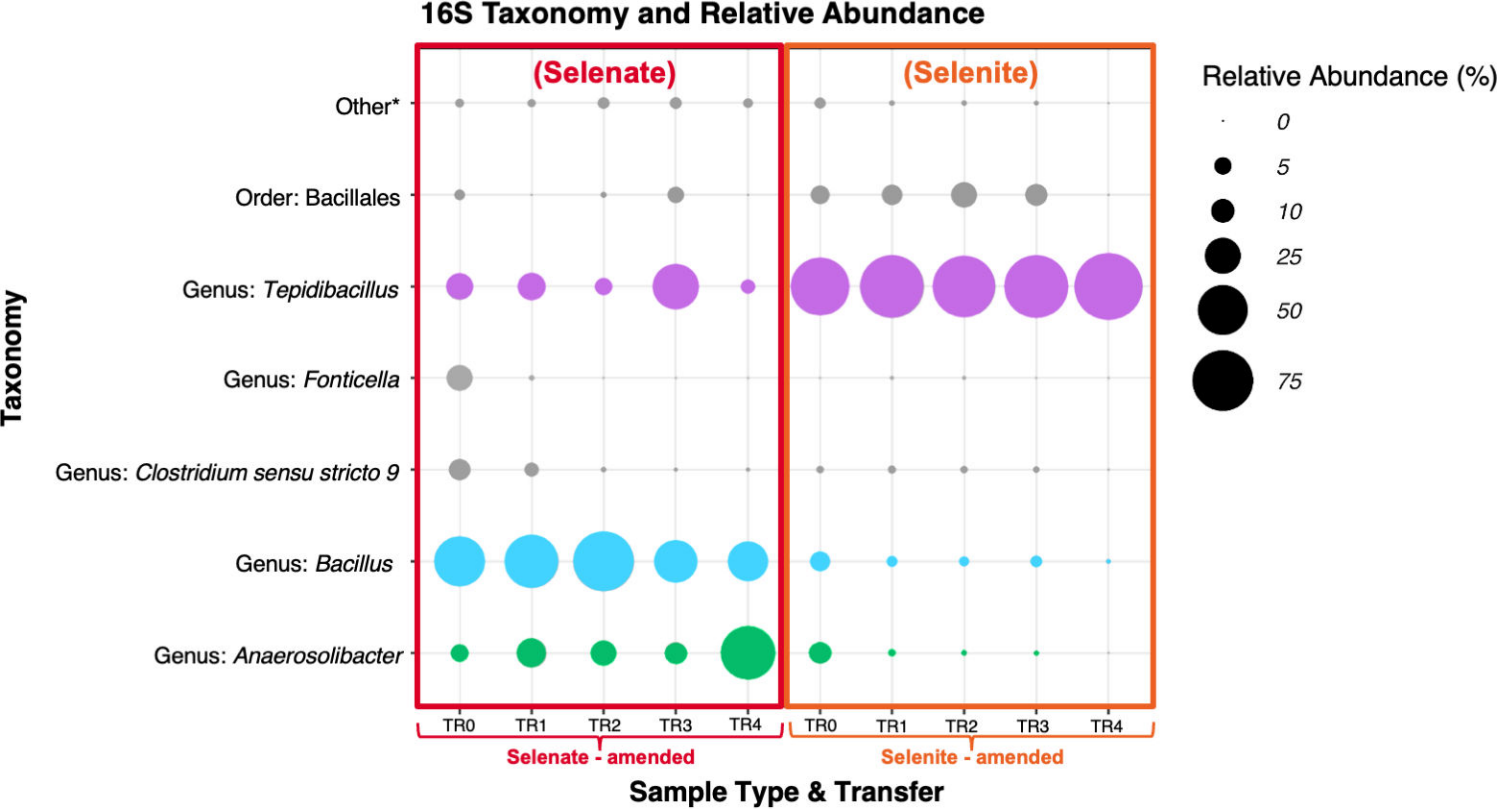
Taxonomy and relative abundance of selenate-amendments and selenite-amendments. Dominant microorganism species change depending on the electron acceptor used, selenate or selenite. Selenate-amended reactors are depicted with a red outline and selenite-amended reactors are depicted with an orange background. Relevant taxa are represented in colored bubbles (purple, blue, and green). Other is composed of all organisms that compose less than 1% of the relative abundance at the species level.

To conduct selection of the SeRB, a minimal salt medium (MSM) was prepared according to Macy et al. (16, 17). Two different selenium-amended reactor types were performed to observe if there were different microbial communities associated with each oxyanion. The media consisted of either a 20 mM selenate-amended MSM or a 20 mM selenite-amended MSM. Selenium oxyanions in the media utilized were about three orders of magnitude greater than what is typically found in FGD wastewater to ensure the selection of the SeRB found in this wastewater. FGD effluent was used as the inoculum for the transfer 0 (TR-0) reactors. Abiotic control reactors with the same media were also run simultaneously without the addition of an inoculum. Red precipitate formed after the incubation period is an indication of formation of elemental (Se^0^), which was typically observed by day 3 of each incubation. Figure 1 depicts the relative abundance up to the genus level via normalized 16S data from the triplicates of each reactor set from each transfer. This study demonstrates two different microbial communities involved in selenate reduction and selenite reduction.

#### Composition of selenate-amended microbial communities

Alpha diversity metrics, Chao1 and Shannon entropy (Table 1), show a drop after TR-0 indicating loss in diversity, which suggests the selection of selenate-reducing bacteria in these samples. From TR-0 to TR-2, biomass increases up to six orders of magnitude higher than the anaerobic community. In reactors TR-3 and TR-4, there is a loss in biomass, but it is still higher than that of the initial community.

Based on the sequencing of the amendments, the major genera providing selenate reduction in FGD fluid were bacteria from the genus *Bacillus*, and a bacteria from the genus *Anaerosolibacter*. The *Bacillus* (metagenomic probing and database corroborates this to be *Mesobacillus* and will be referred to as such going forward) organism had a relative abundance as high as 80% of the microbial community in the selenate-amended reactors but made up ~1.5% of the anaerobic FGD community, and up to ~8% in the selenite-amended reactors. This organism was the predominant organism in the TR-0-TR-2 reactors, making up over 50% of the relative abundance in the reactors. The undefined bacteria from the genus *Anaerosolibacter* represented a relative abundance as high as ~61% of the selenate-amended community but represented ~0.1% of the anaerobic FGD community, and as high as 9% of the selenite-amended community. This organism was the predominant organism in the TR-4 reactors. Another potential selenate reducer may be the bacteria from the genus *Tepidibacillus*, which was found to be most abundant in the TR-3 reactors at 44.6%.

#### Reduction of selenate in selenate-amendments

**Fig 2:**
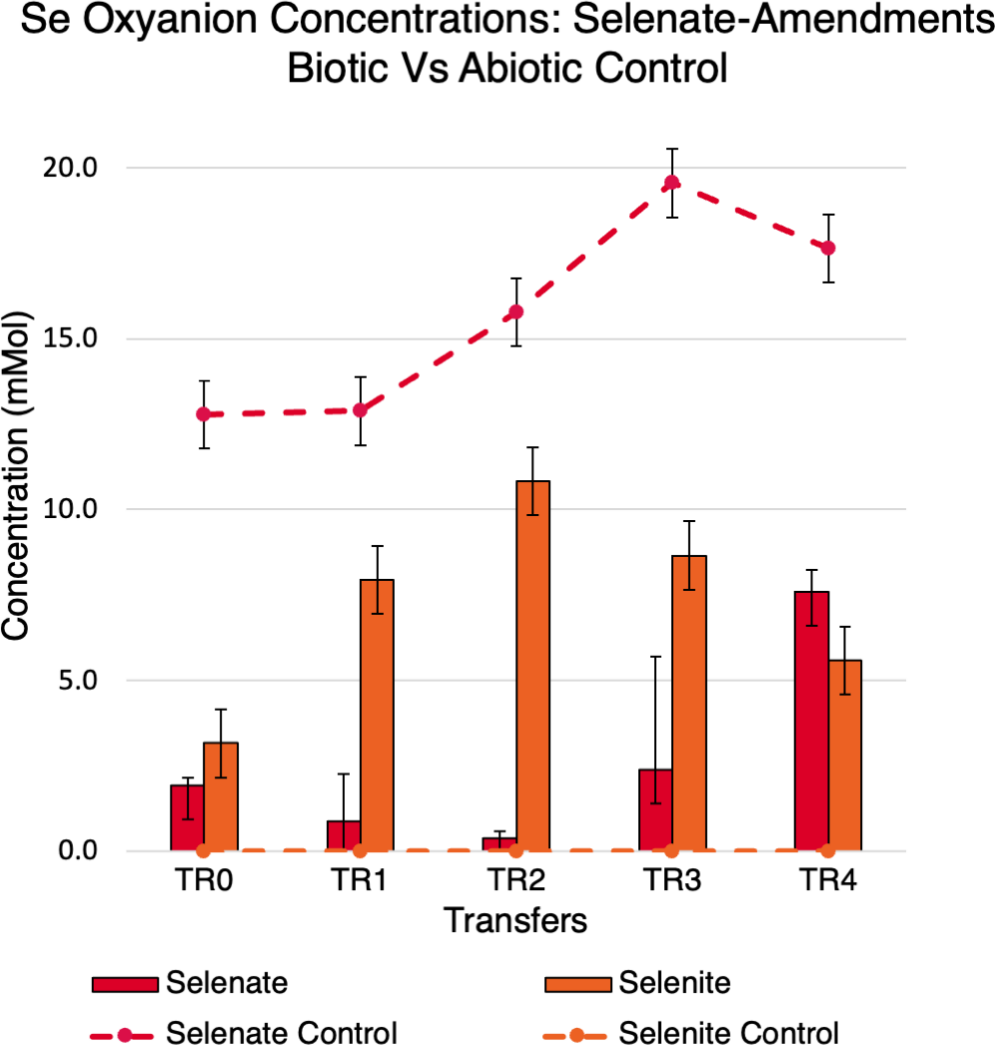
Selenium oxyanion concentrations of selenate reducers. Selenate-reducing microorganisms removed up to 98% of selenate ions (TR-2). Dashed lines are measurements from selenate abiotic control reactors. Bars are measurements from selenate-amendment reactors. Red represents selenate measurements; orange represents selenite measurements.

Selenate reduction was supported by ion chromatography (IC) measurements as depicted in Fig. 2. Both biotic and abiotic control reactor selenium oxyanion concentrations are depicted here. The selenate media contained up to 20 mM of selenate ions and no selenite ions initially. The highest reduction of selenate concentration was observed to be in the TR-2 reactors with a reduction of 98% of the selenate. The lowest reduction of selenate occurred in the TR-4 reactors at 60% removal of selenate. With the reduction of selenate, selenite is produced in the bioreactors in correlation with the amount of selenate removed; with more selenate reduced, more selenite was produced. Selenite production ranged from 3.2 to 10.8 mM in these selenate-amended reactors. The TR-2 reactors had the most selenate reduction and the most production of selenite. The production of selenite agrees with previous studies that show selenate to selenite reduction as a major pathway in the reduction of selenate (4).

#### Composition of selenite-amended microbial communities

The 16S rRNA analysis suggested that the selenite-amended reactors were selected for a selenite reducer. The alpha diversity of these samples was lower than the initial microbial community, which was expected due to the use of a selenite-amended media (Table 1). Biomass is four magnitudes higher than the initial sample in reactors TR-1 to TR-4. However, the TR-0 reactor had biomass that was one order of magnitude lower than the initial sample.

*Tepidibacillus* is the most predominant organism in the selenite-amendment reactors. This organism was in high relative abundance from TR-0 at 74.1% up to 99.8% in TR-4. *Tepidibacillus* only represents ~1.7% of the anaerobic FGD community. Tepidibacillus was also present in the selenate-amended reactors, but at an abundance of 15% or lower in most reactors besides the TR-3 reactors in which it was the most predominant organism in that reactor at that time point.

#### Reduction of selenite in selenite-amendments

**Fig 3:**
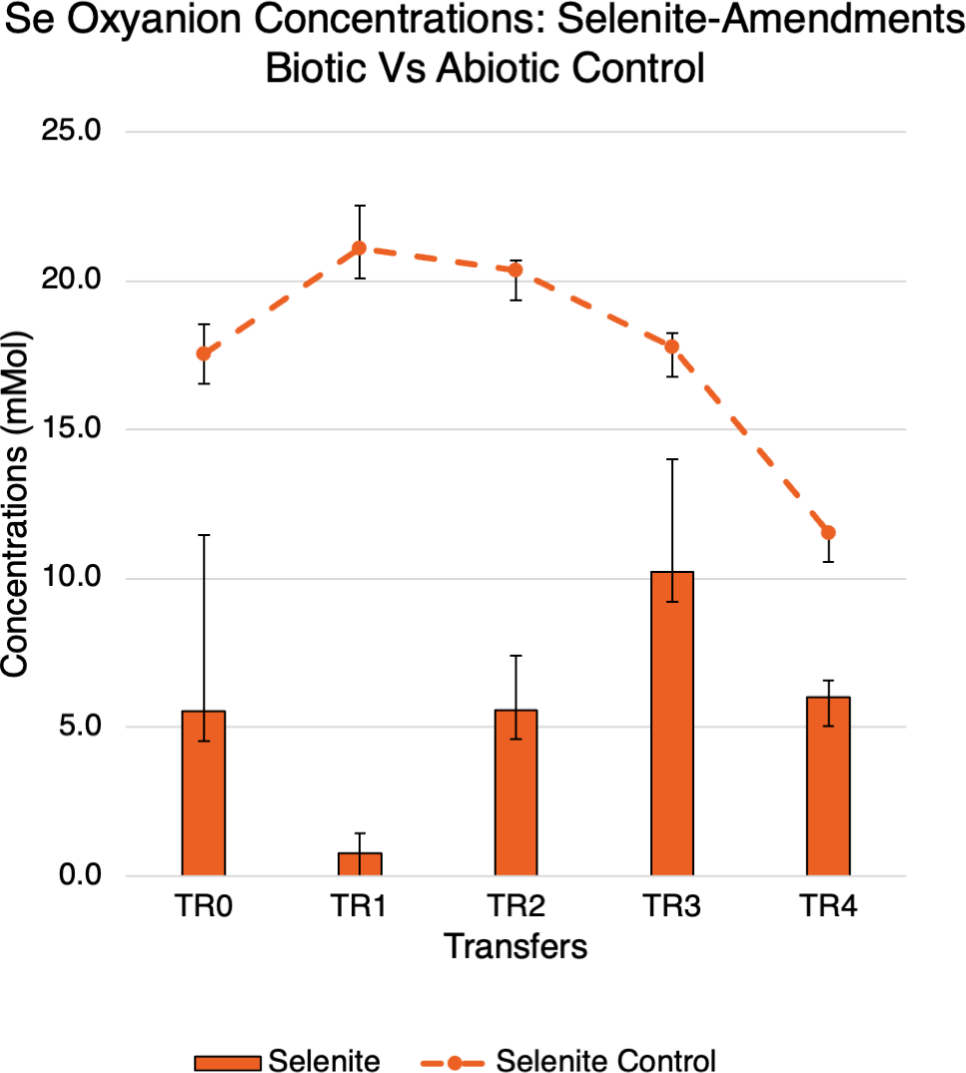
Selenium oxyanion concentrations of selenite reducers. Selenite-reducing microorganisms removed up to 95.1% of selenite ions. The dashed line is measurements from selenite abiotic control reactors. Bars are measurements from selenite-amendment reactors. Red is for selenate measurements; orange is for selenite measurements.

IC analysis also confirmed the reduction of selenite in the selenite-amended reactors as depicted in Fig. 3. Both biotic and abiotic control reactor selenium oxyanion concentrations are depicted here. The initial concentration of selenite in the reactors was ~16 and 0 mM selenate initially (Fig. 3). The highest reduction of selenite was 95% as seen in the TR-1 transfer reactors in comparison to the abiotic reactors. The lowest removal of selenite was 35.9% as depicted by the TR-3 transfer reactor.

### Selenate reduction dependent on microbial community and microbial abundance

**Fig 4:**
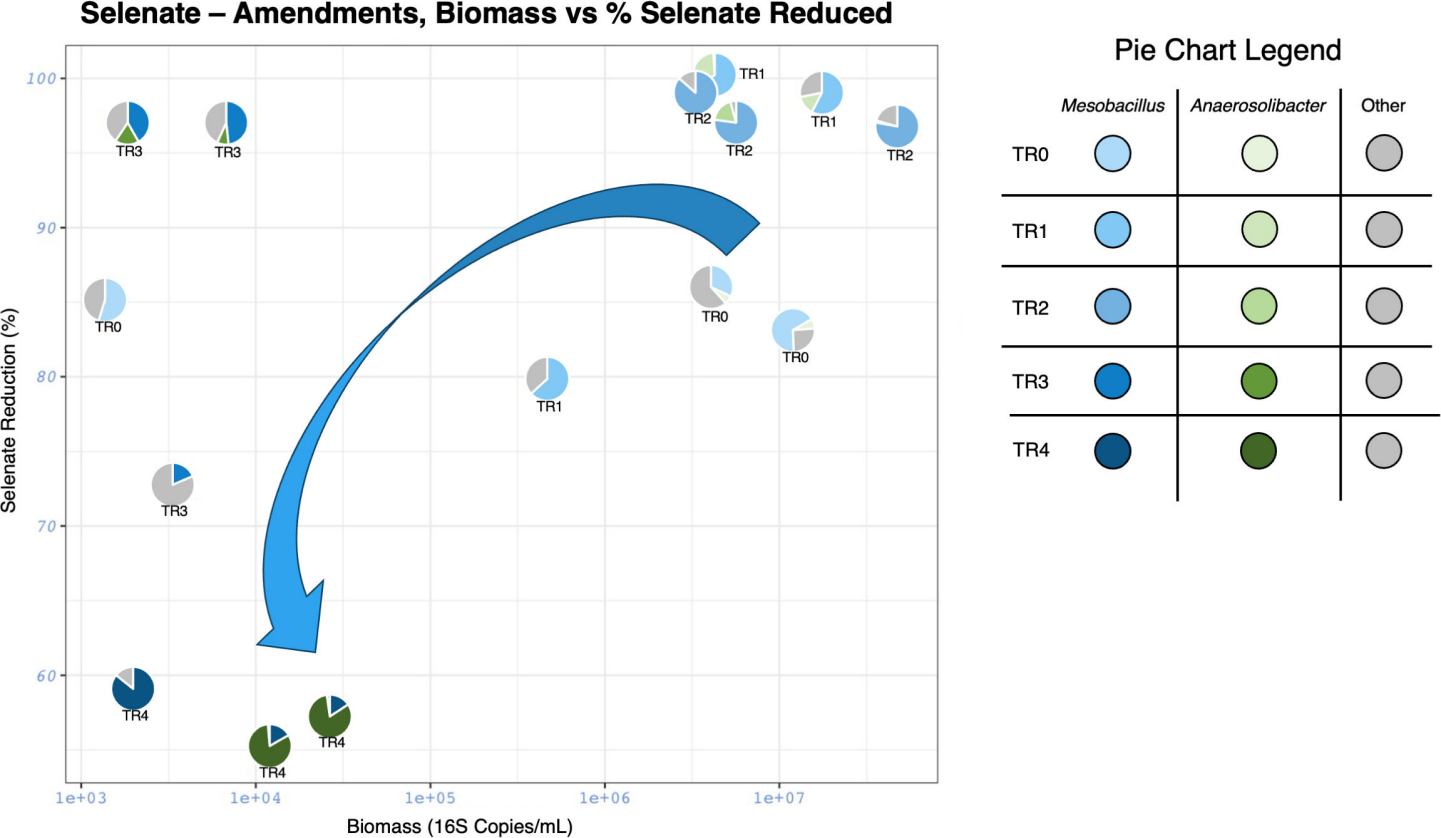
Scatter-pie charts from selenate-amendments. This figure maps percent selenate reduction along the *y*-axis, microbial load along the *x*-axis, and each data point is a condensed pie chart of the relative abundance of the sample. Blue represents *Mesobacillus*, green represents *Anaerosolibacter*. The darker the color, the later the transfer. The blue arrow represents the general trend of biomass with each proceeding transfer.

To better understand the trends observed in the selenate-amendments, percent selenate reduction, microbial load, and relative abundance for all triplicates for each transfer were graphed as a scatter-pie chart, with the relevant organisms denoted and all others considered as “Other” (Fig. 4). The efficiency of selenate reduction increases from TR-0 to TR-3, but then decreases after TR-3. In TR-0 through TR-2, *Mesobacillus* is the predominant organism, comprising over 50% of the relative abundance of the microbial communities. After TR-2, the data suggest a shift in the microbial community with the more noticeable emergence of *Anaerosolibacter* and a loss in microbial load in TR-3 and TR-4 reactors.

The change in percent selenate reduction seems to correlate with both the microbial community and the biomass load. The TR-2 reactors reduced the most selenate on average and had the largest production of selenite. In that reactor, the dominant organism was *Mesobacillus* (79.5%). In the TR-3 reactors, the extent to which selenate was reduced was nearly the same as that in the TR-2 reactors; however, the biomass in these reactors was much less than TR-2. In the TR-3 reactors there were two dominant organisms, *Mesobacillus* (39%) and *Tepidibacillus* (44.6%) on average. There seems to be a rise of *Tepidibacillus* because there was a high production of selenite in the TR-2 reactors, which also suggests that selenite may affect *Mesobacillus* negatively as there was a loss in its relative abundance in TR-3. *Tepidibacillus* seems to be slow growing because it was more predominant in the TR-3 reactors than the TR-2 reactors, where there was more selenite available. This organism also seems to be selenate-tolerant as it can survive in an environment with high selenate concentration in contrast to *Mesobacillus* and *Anaerosolibacter* which are in low abundance (less than 10%) in the selenite-amended reactors, suggesting that these organisms are not selenite-tolerant. The TR-4 reactors had lower selenate reduction than the prior transfers. In this microbial community, the dominant organisms were *Anaerosolibacter* (61.5%) and *Mesobacillus* (33.6%) on average. *Anaerosolibacter* seems to be a slow grower as it has been present in the other transfers, but it was not as dominant. Selenite may be toxic to *Mesobacillus* because its relative abundance fell after the largest production of selenite seen in TR-2 reactors, and it makes up a very small percentage in the initial selenite-amendment reactors. In addition, the microbial load also seems to correlate with the reduction of selenium, where the load was higher in TR-0, TR-1, and TR-2 reactors.

The dominant microorganisms were *Mesobacillus* and *Anaerosolibacter*, suggesting these were selenate-reducing microorganisms, while the later emergence of *Tepidibacillus*, and the presence of *Tepidibacillus* in the selenite-amended reactors suggests that this organism emerged more with the production of selenite.

#### Loss in efficiency of selenite reduction with proceeding transfers

**Fig 5:**
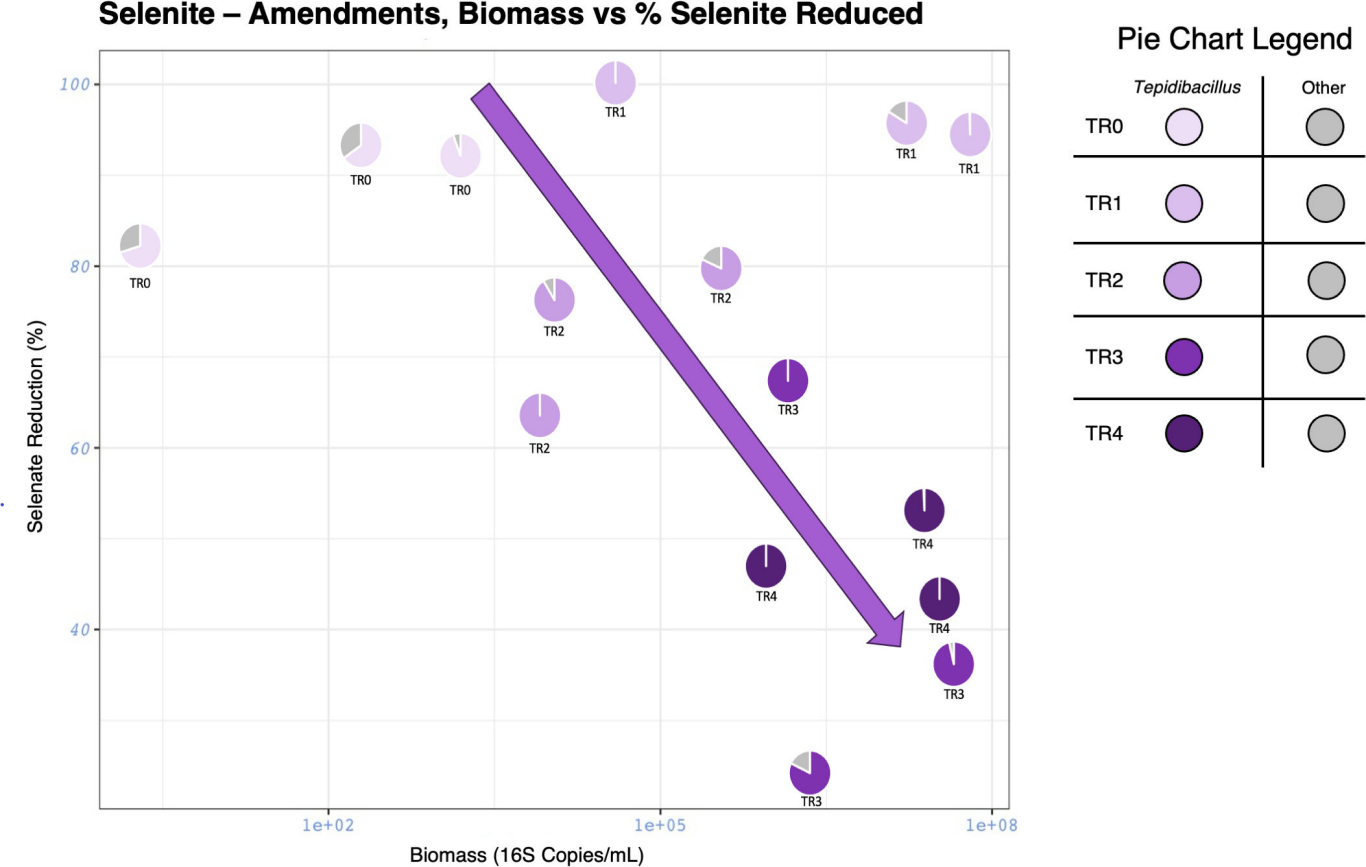
Scatter-pie charts from selenite-amendments. Map of percent selenate reduction versus microbial load for each transfer. The pie chart for each data point is a condensed relative abundance of the sample. Purple represents *Tepidibacillus*. The darker the color, the later the transfer. The purple arrow represents the general trend of biomass with each proceeding transfer.

To better understand the trends in the selenite-amendments, percent selenite reduction, microbial load, and relative abundance for all triplicates for each transfer were assessed (Fig. 5). *Tepidibacillus* becomes the predominant organism in all the reactors, further suggesting this microorganism is being selected for in both the selenate and selenite reactors for its selenite reduction functional potential. There is a loss in selenite reduction efficiency with each transfer despite the microbial load and selection for *Tepidibacillus* increasing with each transfer.

Selenite reduction did not correlate to the microbial community structure or biomass load; however, there seems to be a loss in selenite reduction with each proceeding transfer. The TR-1 reactors reduced the most selenite on average, where the predominant organism was *Tepidibacillus* (88.2%) and had Bacillales (8.1%) as the second most abundant organism. The extent of selenite reduction fell in the reactors following the TR-1 reactors, despite an increasing biomass and that *Tepidibacillus* remained the dominant organism, as high as 99.8% in TR-4 reactors. One potential reason is the lack of microbial diversity, leading to the lack of exchange in metabolites that would trigger selenite reduction in *Tepidibacillus*. The exchange of metabolites is gaining increasing recognition as a pivotal aspect of microbial cell physiology within community frameworks. The absorption and secretion of metabolites are fundamental traits of metabolism, fostering cross-feeding and collective survival of the whole microbial community (18–21).

### Metagenome assembly and draft genome recovery

After quality trimming and assembling the generated metagenomic sequences, three metagenome-assembled genomes (MAGs) were recovered from three different samples representing two from the selenate-amendments (TR-2 and TR-4 reactors) and one from the selenite-amendments (TR-4 reactor). Completeness of the recovered MAGs ranged between 87.68% and 99.43%, while contamination ranged between 1.18% and 4.95%. Taxonomic annotation using the applied maker gene sets in CheckM allowed the assignment of the recovered MAGs to the following taxa: Bacillaceae, Clostridiales, and Paenibacillaceae; and GTDB-tk allowed the assignment of the recovered MAGs to the following genera: *Mesobacillus*, *Anaerosolibacter*, and *Tepidibacillus*. These genera were also present at high abundances in the amendment samples based on the 16S sequencing data.

The MAGs recovered were representative of the most abundant organisms based on 16S sequencing. Time points TR-2 and TR-4 from the selenate-amendments were used for metagenomic sequencing, in which, one high-quality MAG was recovered from each sample. Kaiju determined that the TR-2 reactor was mostly representative of *Bacillus selenatarsenatis* with ~77% of the reads classified, which was corroborated with GTDB-tk where the closest relative to the MAG in TR-2 was *Mesobacillus selenatarsenatis* in which the closest placement average nucleotide identity was 91% similar. *B. selenatarsenatis* is a basionym of *M. selenatarsenatis* (22). *Mesobacillus* organisms are classified as rod-shaped cells that are gram-positive or gram-variable in staining. These organisms come from varied environments including soil, human gut, groundwater, and wastewater (23). *M. selenatarsenatis* is a facultative anaerobe that utilizes selenate and arsenate and was isolated from an effluent drain of a glass manufacturing plant (24). This organism was first found in 2001 and studied further by some of the same authors in 2007 (24, 25). This bacterium was reported to have stained gram-positive, which is notable because this genus is also known for gram-variable organisms (23, 24). Because the *Mesobacillus* identified here had only a 91% average nucleotide identity to that of *M. selenatarsenatis*, it is likely that the *Mesobacillus* in this study is a novel species.

For the TR-4 reactor, Kaiju determined the closest relative is *Thermotalea metallivorans* with ~77% of the reads classified, while GTDB-tk determined the closest relative to be *Anaerosolibacter* carboniphilus, where the closest placement average nucleotide identity was 81%. This species is a strictly anaerobic organism that utilizes a range of carbohydrates and organic acids (26). However, it does not reduce sulfate, thiosulfate, nitrate, or nitrite (26). Because only a fraction of the reads were classified and this species did not correlate to that of the 16S, it is unlikely that this species is representative of what was identified as *Mesobacillus. Anaerosolibacter* is a recently identified genera. They are described to be straight, rod-shaped organisms that are gram-negative (27). *A. carboniphilus* is known to be a strict anaerobe that utilizes carbohydrates, elemental sulfur, thiosulfate, and sulfate as electron acceptors (27). The *Anaerosolibacter* organism identified in the amendment experiments in this study is a novel species as the average nucleotide identity was only 81% when compared to *A. carboniphilus. A. carboniphilus* is the only other defined bacterium of this genus (27). This study is the first to suggest *Anaerosolibacter* may have selenium-reducing capabilities.

Reactor TR-4 from the selenite-amendments was used for metagenomic sequencing, in which one high-quality MAG was recovered to 88% completeness. This MAG was representative of the most abundant organism based on the 16S sequences. Kaiju determined that the sequences were similar to that of *Tepidibacillus decaturensis*, where ~80% of the reads were classified. GTDB-tk determined that the MAG was also similar to that of *T. decaturensis*, where the average nucleotide identity was 98% similar. This species is a microaerophilic organism that is known to utilize a broad range of organic and inorganic substrates (28). If this organism is indeed *T. decaturensis*, this study would be the first to report that this organism has the ability to utilize selenite (28). The only other *Tepidibacillus* strain found to utilize selenium oxyanions is *Tepidibacillus infernus*, which is an aerotolerant anaerobe that can utilize both selenate and selenite (29).

### Known selenium oxyanion reduction genes matched in MAGs

**Fig 6:**
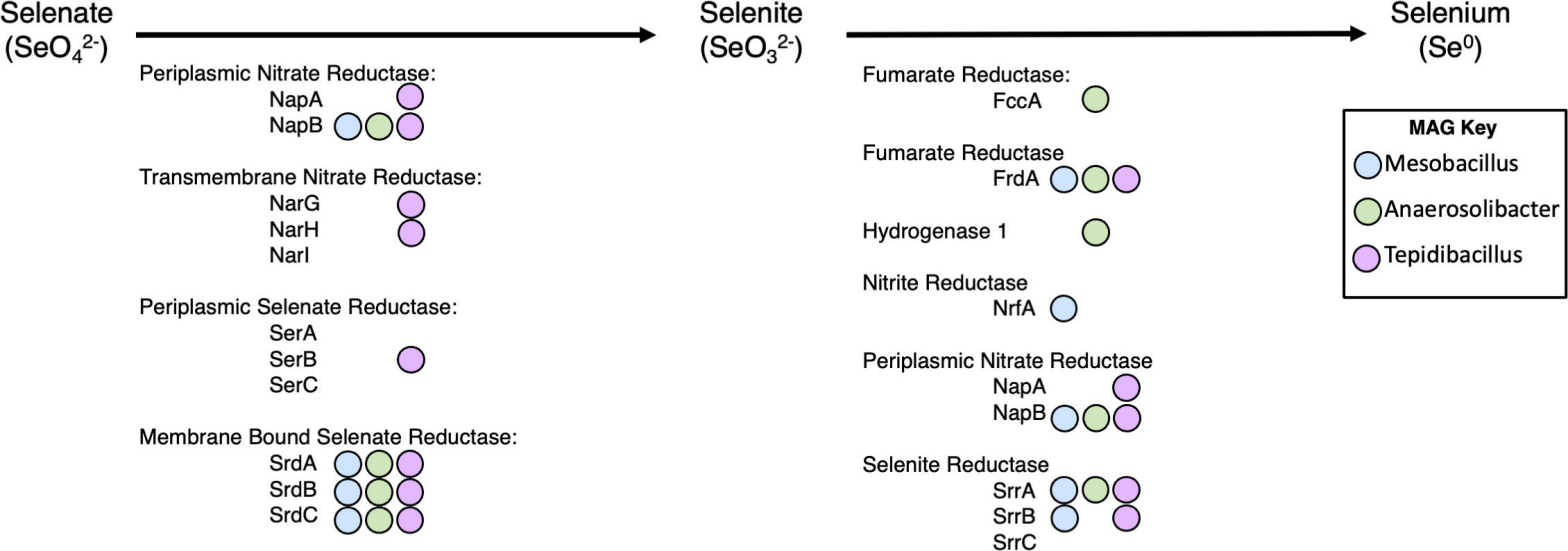
Condensed selenium reduction reaction and relevant associated genes. Biological reduction of selenium occurs in two steps: first with the reduction of selenate to selenite, and second selenite to elemental selenium. Relevant selenium reduction genes are denoted under the associated reaction. Presence of relevant genes is denoted with colored circle above gene abbreviation.

The MAGs recovered were evaluated for the presence of 48 known selenium oxyanion reduction genes to enhance understanding of the significance of individual selenium-reducing bacteria species in FGD wastewater (30). Figure 6 illustrates the transformation of selenate to selenite and then to elemental selenium, with relevant genes and MAGs indicated by colored dots.

Relevant selenate reduction proteins were reported to be periplasmic nitrate reductase (NapA and NapB), transmembrane nitrate reductase (NarG, NarH, and NarI), periplasmic selenate reductase (SerA, SerB, and SerC), and membrane-bound selenate reductase (SrdA, SrdB, and SrdC). Relevant selenite reductase proteins were reported to be fumarate reductase (FccA and FrdA), hydrogenase 1, nitrate reductase (NrfA), periplasmic nitrate reductase (NapA and NapB), and selenite reductase (SrrA, SrrB, and SrrC).

Periplasmic nitrate reductase was reported to have selenate reduction ability in *Rhodobacter spaeroides* and selenite reduction ability in *Shewanella oneidensis* MR-1 (4, 8). In the MAGs evaluated, NapA was present in *Tepidibacillus*, and NapB was present in all three MAGs, suggesting involvement in selenate reduction with potential organism-specific encoding. Transmembrane nitrate reductase from *Escherichia coli* shows *in vitro* selenate reduction ability. NarG and NarH were present in the *Tepidibacillus* MAG. NarI was not present in any of the MAGs. This suggests that *Tepidibacillus* may be utilizing both periplasmic and transmembrane nitrate reductases for selenite reduction.

Two proteins, periplasmic selenate reductase (SerA, SerB, and SerC) and membrane-bound selenate reductase (SrdA, SrdB, and SrdC), were examined. Periplasmic selenate reductase from *T. selenatis* and membrane-bound selenate reductase from *B. selenatarsenatis* strain SF-1 has been extensively characterized (8, 31, 32). *Tepidibacillus* is the only MAG that contained the periplasmic selenate reductase (SerB), suggesting that this organism can perform selenate reduction or is using this protein for selenite reduction and that this reaction may be occurring in the periplasm. All three MAGs contained membrane-bound selenate reductase for all of SrdABC. The presence of these genes in *Mesobacillus* and *Anaerosolibacter* indicates an ability to respire selenate, with Tepidibacillus potentially less efficient in selenate reduction, reflected by its lower abundance in the selenate-amended reactors. The low presence of *Tepidibacillus* in the selenate-amendments and high presence in the selenite-amendments suggests that this organism may use its membrane-bound selenate reductase for selenite reduction.

Fumarate reductase (FccA) in *S. oneidensis* MR-1 has been shown to be involved with anaerobic selenite reduction (8, 10). This was matched to the *Anaerosolibacter* MAG, suggesting it may be utilized in selenate reduction. *Anaerosolibacter* composes less than 1% of the relative abundance in the selenite-amended reactors proceeding with the TR-0 reactors. In contrast, fumarate reductase (FrdA) from *Enterobacter cloacae* Z0206 is matched in all three MAGs. This is interesting as this protein was found to be involved in selenite reduction aerobically. This suggests that organisms represented by these MAGs may be aerotolerant in a microaerophilic environment.

Hydrogenase 1 from *Clostridium pasteurianum* has been reported to support selenite reduction (4). This was matched to *Anaerosolibacter* MAG, suggesting it may be involved in selenate reduction as the data suggest that this organism is not utilizing selenite.

Nitrite reductase (NrfA) from *S. oneidensis* MR-1 has been shown to be involved with anaerobic selenite reduction (4, 8). This was matched to the *Mesobacillus* MAG, suggesting this protein be utilized in selenate reductase as this was one of the predominant organisms in the selenate-amended reactors.

Selenite reductase in *Bacillus selenitireducens* MLS10 was recently identified (33). The SrrA subunit was mapped to all three MAGs and the SrrB subunit was mapped to the *Mesobacillus* and *Tepidibacillus* MAGs. None of the MAGs mapped to the SrrC subunit. The presence of these genes in the *Mesobacillus* and *Anaerosolibacter* MAGs suggests that these organisms may be able to utilize selenite slightly, or that they are using it for selenate reduction, or that what they encode for the SrrC subunit is different than what is currently annotated. The presence of these genes in the *Tepidibacillus* MAG is evidence that this organism is capable of respiring selenite.

### Selenium bioparticles increase in purity with proceeding transfers

**Fig 7:**
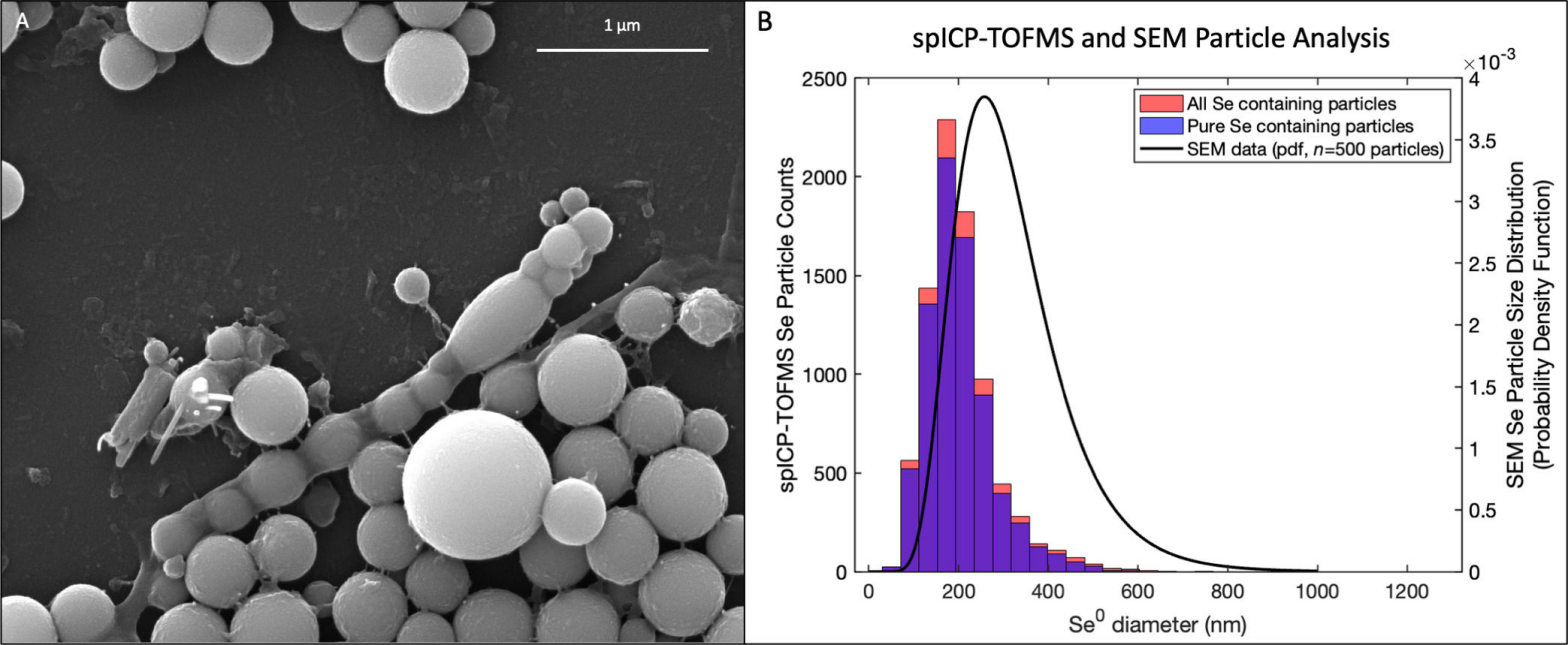
SEM and spICP-TOF-MS data of elemental selenium nanoparticles (SeNP) and sub-micron particles produced by selenite reducers. SEM image, magnification 59,394×, showing Z-contrast of what looks like to be intracellular selenium particles produced by selenite reducers. This particular image is from the selenite-amended reactors. Five hundred particles from SEM images were analyzed and plotted with spICP-TOF-MS data. TOF detected 8228 selenium-containing particles, most of which were largely purely selenium.

SEM imaging of the solids of these reactors showed evidence of the production of biogenic elemental selenium particles and was able to capture microbial-like structures (Fig. 7A). EDS spectra analysis confirmed that the particles are composed largely of selenium (S3). ImageJ analysis of the particles shows that the average size of these particles from the selenate-amended reactors is ~300 nm, while those from the selenite-amended reactors are ~400 nm. Size analysis of particles from spICP-TOF-MS data showed that selenium particles were slightly smaller on average than reported with SEM. XRD analysis of these samples showed that these particles were composed of amorphous selenium as well as hexagonal selenium (S4). spICP-TOF-MS analysis showed that the majority of the particles were pure elemental selenium without entrained metals (Fig. 7B). Bioproduced selenium increases in purity with each proceeding reactor for both selenate-amended and selenite-amended reactors (S5). This suggests that FGD WW can be a potential source for selenium resource recovery with little need for post-processing.

### Location of formation biogenic selenium remains unclear

SEM imaging showed some evidence of microbial-like structures; however, it was unclear with both the images and metagenomic data where the biogenic selenium was being formed. Some images captured seem to suggest that the production of nanospheres is intracellular based on the bacteria-like structures imaged, but future work must be completed to confirm if the nanoparticles are being formed in the membrane, within the cytoplasm, or extracellularly.

The primary difference between the selenate-amended reactors that produced 300 nm nanospheres and the selenite-amended reactors that produced 400 nm nanospheres was the microbial community. This is a known idea, where the species of the organism affects the formation and structure of the elemental selenium formed (34). The biogenic selenium from both reactor types was found to get as large as a micron in diameter as shown by SEM imaging; interestingly, spICP-TOF-MS was not able to capture evidence of particles of this size. Finding particles as large as a micron is interesting because much of the genes identified for selenate or selenite reduction was denoted to be found in the periplasm (NapA, NapB, SrdA, SrdB, SrdC, SrrA, SrrB, and SrrC) (4, 8, 33). The periplasm is known to be 10–50 nm in thickness, which suggests that there may be a transport mechanism involved in shuttling the elemental selenium out of the cell once it is formed (8, 35–37).

Identifying the factors that govern particle size can lead to the engineering of bioreactors with ideal parameters to produce particles of precise dimensions. Enhanced control of elemental selenium particle size could yield utility savings in water treatment, particularly in ultrafiltration as a polishing step if the particle sizes were larger. If the particle sizes were manipulated to be smaller, the utility may be able to recover and sell the particles for use in industries such as nanomedicine, environmental remediation, and electronics (7).

### Conclusions

This work is the first to report on the culturability, taxonomic identification, and metabolic insights of the anaerobic SeRB in FGD wastewater. Three MAGs were recovered, revealing the presence of *Mesobacillus*, *Anaerosolibacter*, and *Tepidibacillus*. These species are associated with selenate or selenite reduction, with evidence suggesting the identification of novel species of *Mesobacillus* and *Anaerosolibacter*. Notably, *Tepidibacillus* was shown to respire selenite, making it a new discovery for this organism. Despite an increased microbial load and the dominance of *Tepidibacillus*, selenite reduction efficiency declined with each transfer, likely due to a decrease in microbial diversity that limited metabolite exchange. MAGs contained several important genes for selenium reduction such as NapB, SrdABC, and SrrA. *Tepidibacillus* demonstrated potential for both selenate and selenite reduction, while *Mesobacillus* and *Anaerosolibacter* contributed primarily to selenate reduction, with potential pathways identified for each organism. SEM suggested the formation of intracellular elemental selenium particles; however, the exact location of particle formation remains to be determined. Elemental selenium particle purity increased with each reactor transfer, suggesting that FGD wastewater can be a viable source for selenium recovery with minimal post-processing required. These findings highlight the potential for optimized biotreatment strategies to enhance bioremediation and resource recovery from FGD wastewater, offering both environmental and economic benefits. As the US energy landscape evolves, such strategies promise cleaner and healthier waterways and more sustainable industrial practices.

## MATERIALS AND METHODS

### Field sampling

Samples for amendment reactors were sourced from a coal-fired powerplant’s FGD WW treatment system in the Appalachian region. Fluid sample was collected from the alternative discharge tank (ADT) for wastewater from the FGD system. The sample was collected in a sterilized Nalgene bottle that was filled completely with no headspace and sealed to maintain anoxic conditions. The sample was immediately stored at −20°C to minimize microbial activity and changes to the microbial community.

### FGD aerobic and anaerobic microbial community studies

During processing, FGD wastewater stratifies such that most of the solids sink to the bottom of the bottle. 50 mL of the stratified water was taken in triplicate from the most sediment-free sections and subjected to 16S sequencing and geochemical processing, referred to as “Aerobic FGD.” The microbial community studied here is thought to be primarily of aerobic microorganisms. Water from field sampling was filtered and then DNA was extracted from the filters and amplified for the 16S region and subsequently sequenced. This microbial community is depicted in Fig. S1.

To get a better resolution of the anaerobic FGD microbial community, 50 mL of shaken FGD water (in triplicate) was subjected to anaerobic conditions and incubated for 7 days at 40°C, mimicking FGD conditions. These samples are referred to as “anaerobic FGD.” Samples were filtered, DNA extracted, 16S region amplified, and then sequenced much like the initial community.

### Selection of selenium oxyanion reducing bacteria

Growth media that were used to enrich SeRB are based on Macy et al. (16). MSM contained (g L^−1^; pH ~7–7.2): NaCl, 1.2; KCl, 0.3; NH_4_Cl, 0.3; KH_2_PO_4_, 0.2; Na_2_SO_4_, 0.3; MgCl_2_ · 6H_2_O, 0.85, CaCl_2_ · 2H_2_O, 0.1. The following were added per liter of MSM: 1 g NaHCO_3_, and 1 g yeast extract. The following were added to the MSM: 1 mL of trace elements solution SL-10 (FeCl_2_ · 4H_2_O, 1.5; CoCl_2_ · 6H_2_O, 0.19; MnCl_2_ · 4H_2_O, 0.1; ZnCl_2_, 0.07; Na_2_MoO4 · 2H_2_O, 0.036; NiCl_2_ · 6H_2_O, 0.024; H_3_BO_3_, 0.006; CuCl_2_ · 2H_2_O, 0.002; HCl [25%] 0.01 mL), 10 mL of ATCC vitamin solution. The following stock solutions were added to the medium to final concentrations of 20 mM of acetate and either 20 mM (189,000 µg/L) of sodium selenate or 20 mM (173,000 µg/L) of sodium selenite before adjusting pH to 7–7.2. Concentrations of the oxyanions added are about three magnitudes higher than what is typically found in FGD wastewater. 45 mL of the medium was dispensed into sterile 60 mL serum bottles. The serum bottles were sealed with rubber stoppers and held in place by aluminum crimps and then sparged with 100% N_2_ for at least 30 min to ensure anoxic conditions. 5 mL of homogenized ADT was used to inoculate for SeRB. Reactors were sparged with 100% N_2_ for at least 20 min after inoculation. Cultures were then incubated in the dark for 7 days at 40°C at 100 rotations per minute (rpm) (Innova 44, New Brunswick Scientific). Reactor sets without FGD fluid were used as abiotic controls to assess the influence of any abiotic factors in the reduction of the selenium oxyanions. Reactor sets without selenium oxyanions were used to confirm no spontaneous reduction of selenium oxyanions.

Qualitative screening was performed by monitoring the first occurrence of red precipitates. Observation of red precipitates is an indication that either the selenate [Se(VI)] or selenite [Se(IV)] contained in the anaerobic amendment medium was biologically reduced to insoluble elemental selenium [Se(0)]. After the incubation of FGD inoculation in selenium oxyanion enriched media, samples were transferred to fresh growth media as described above. Cultures were incubated at 40°C at 100 rpm in the dark and transferred four times over a total period of 45 days to further enrich the community with the ability to reduce selenate or selenite. Each transfer was over a period of 7 days. Media samples were collected before inoculation and culture broth samples were collected at the end of each transfer. Controls consisted of three 50 mL samples of FGD fluid as well as 50 mL of each growth media. This paper covers chemical analysis for all transfers (TR-0 to TR-4).

### Selenium oxyanion reduction by amendment bioreactors

The amendments were used to establish the extent of selenate or selenite reduction of a selenium species-rich media (either selenate-amended or selenite-amended) and to confirm that these organisms existed in the wastewater of the FGD system. Amendments and each transfer were done in triplicate and steps were taken to ensure all reactor types received the same type and amount of selenium oxyanion reducing inoculum. Cultures and controls were all incubated at 40°C at 100 rpm in the dark. Each transfer was incubated for 7 days to ensure sufficient time for microbes to reduce selenium oxyanions. Each transfer type is indicated with the following notation: TR-1 (first transfer after initial inoculation), TR-2 (second transfer using TR-1 as inoculum), TR-3 (third transfer using TR-2 as inoculum), and TR-4 (fourth transfer using TR-3 as inoculum). A visible red precipitate is an indication of the production of elemental selenium (Se^0^).

### Analytical procedures

For each transfer, 5 mL of incubated culture samples was used for inoculation. The remaining sample (~45 mL of culture) was filtered through a 0.20 µm polyethersulfone filter (Pall Corporation, Port Washington, NY, USA) to exclude biomass and any other suspended solids. Filtered samples were stored at −20°C for SEM and DNA analysis. 15 mL of filtrate was filtered a second time through a 0.22 µm sterile polyethersulfone filter (MilliporeSigma). 10 mL of the filtrates was assessed with IC and 5 mL of the filtrates were used for pH measurements.

### SEM

Scanning electron microscopic studies were performed on the processed samples. Sample processing consists of harvesting, washing, fixing, and drying of cells and cellular material. Cells were harvested by transferring thawed samples from the filter cake from the 0.2 µm Pall Corporation filter using sterile clay sculpting tools onto poly-l-lysine coated glass coverslips. Harvested material was washed thrice with phosphate-buffered saline (PBS, pH 7.4). Fixation was done with modified Karnovsky’s fixative (2% paraformaldehyde and 2.5% glutaraldehyde in a 0.1 M sodium phosphate buffer, pH 7.4). Harvested material was washed again with PBS. Material was then dehydrated through a series of alcohol dehydration steps (30%, 50%, 70%, and 100%). After dehydration, t-butyl alcohol was layered onto the samples. Finally, hexamethyldisilazane (HMDS) was used to dry the samples. Samples were mounted onto pin stubs and sputtercoated with 4 nm Au-Pd coating. Samples were imaged using an FEI Quanta 600F SEM for physical topography and atomic number (Z) contrast under high vacuum conditions. For all SEM analysis, a beam voltage of 20 kV and spot size of 3.0 was used. Energy dispersive spectroscopy (EDS) was performed using Oxford Instruments’ INCA x-act detector for semi-quantitative elemental analysis. Particle size distribution was accomplished using SEM images and analyzing 500 particles by using the software ImageJ (38).

### IC

Major anion species were determined by IC with a Thermo Scientific Dionex ICS-5000+. Anions were quantified by conductivity detection with electrolytically regenerated suppression using Dionex AERS 500-4 mm and carbonate removal with Dionex CRD 200-4 mm. IC species separation was done using an AS11-HC 4 × 250 mm^2^ column at 30°C. Selenium anion species (selenate and selenite) were determined using Dionex EGC III KOH eluent at constant 20 mM concentration. Other major anions were determined using a gradient KOH concentration of 1–60 mM. All samples were run in duplicate and anion control samples were analyzed every 10–15 samples. The inorganic anion control standard was a Sigma-Aldrich multianion standard, and the organic anion control standard was prepared at two ppm from Sigma-Aldrich anion standards. Autoclaved and filter-sterilized 20 mM of sodium selenate and 20 mM sodium selenite solutions were made as selenium standards. Anion control standards were generally within 90–110% accuracy.

### XRD

Samples were analyzed on a PANalytical X’Pert Pro or on a PANalytical EMPYREAN diffractometer. On the X’Pert Pro equipped with Cu Kα radiation and Xcelerator parallel plate detector, cellular material from filters was harvested and transferred to a zero-background silicon wafer holder with a sponge tip brush. The sample was then mounted onto a spin mount to ensure even distribution during analysis. Data were collected over a 2*θ* range of 5–75°, with a step size of 0.03° and a counting time of 500–100 s per step. On the EMPYREAN, cellular material was loaded onto the sample holder with a sponge tip brush. Data were collected over a 2*θ* range of 5–75°, with a step size of 0.05° and a counting time of 500–100 s per step.

### spICP-TOF-MS

Cellular material composition was determined with spICP-TOF-MS. Sample processing consisted of harvesting cellular material from filters and suspending ~1 µg in 15 mL diH_2_O and immediately analyzed by spICP-TOF-MS. The instrument operated in oxygen reaction mode. Dissolved multielement solutions were used as calibrations between 0 and 20 ppb were used to quantify the particles in each measurement. A 40 nm Au NP standard solution (nanoComposix) was used to determine the transport efficiency (39). The nebulizer gas flow rate, nebulizer liquid flow rate, attenuated masses, TOF extraction frequency, cooling gas flow rate, and plasma power are reported in another recent study (40). The nebulizer gas flow rate, nebulizer liquid flow rate, attenuated masses, TOF extraction frequency, cooling gas flow rate, and plasma power are reported in another recent study (41). An integration time of 0.002 s was used to identify individual particle events (41). To account for large particle sizes, up to three consecutive events were merged into one. All spICP-TOF-MS data were processed with TOFPilot software. The format of the single-particle data were given in time series for all of the measured isotopes in csv format. Post-processing analysis was performed in Python.

Samples were introduced using a peristaltic pump (Thermo-Fisher) mounted to the ICP-TOF, an aerosol was generated by a MicroMist nebulizer and a cyclonic spray chamber cooled to 4°C. The sample flow rate was 0.4 mL/min. A 2.5 mm injector set to 5 mm sampling depth was used. Ni cones were used. Samples were recorded for 360 s with dwell times of 2 ms. Dissolved calibration standards were recorded for 60 s with dwell times of 1 s. Calibrations were conducted between 0.01 and 20 ppb with eight calibration points.

### DNA extraction

Samples from Triplicates 1 and 2 were extracted by using a modified protocol of the DNeasy Powersoil Pro Kit (Qiagen, Hilden, Germany). Approximately 45 mL of culture were filtered through 0.20 µm polyethersulfone filter (Pall Corporation, Port Washington, NY, USA). Triplicate 1 filtered samples were cut into quarters. DNA was extracted from one quarter of the filter with collected biomass by following the protocol of the kit. For triplicate 2, the whole filter with the collected biomass was put into a 5 mL microcentrifuge tube and two bead tubes, and 1,200 µL of CD1 was utilized for the initial steps of the protocol. Once the sample had been vortexed, the extraction was performed in duplicate for each sample until it was time to combine the samples during the spin filter step.

Samples from Triplicate 3 were extracted by using a modified protocol of the DNeasy Powersoil Kit (Qiagen, Hilden, Germany). Approximately 45 mL of culture were filtered through a 0.20 µm polyethersulfone filter (Pall Corporation, Port Washington, NY, USA). The filter with the collected biomass was put into a 5 mL microcentrifuge tube and incubated with 20 µL of 20 mg/mL lysozyme (Sigma Aldrich) at 37°C for 30 min. Following the lysozyme incubation, the extraction was then performed using the recommended guidelines in the manufacturer’s protocol. 100 µL of DNA was eluted from the spin filters of the kit. Kit blanks were done with each extraction set.

### Absolute quantification (ddPCR) of DNA

Extracted DNA was quantified utilizing a QX200 Droplet Digital PCR System (BioRad, Hercules, CA, USA). In droplet digital PCR, DNA samples and the ddPCR EvaGreen Mastermix are divided into about 20,000 droplets which are then amplified using custom 16S rRNA gene primers (F: GTGSTGCAYGGYTGTCGTCA; R: ACGTCRTCCMCACCTTCCTC) with an expected amplicon size of 146 base pairs (42).

### 16S rRNA library preparation and sequencing

Extracted DNA and kit blanks were amplified using universal primers targeting the V4 region of the 16S RNA gene, as previously described in reference 43. Each sample had eight technical replicates pooled to obtain enough PCR product. Pooled PCR products were cleaned using SPRIselect beads (Beckman Coulter, Pasadena, CA, USA) and visualized on the Agilent Bioanalyzer using the High Sensitivity Assay Kit (Life Technologies, Santa Clara, CA, USA). Negative PCR controls were also amplified and visualized in order to confirm no contamination occurred. After visualization, successful PCR products were quantified using the Qubit dsDNA High Sensitivity Assay Kit (Life Technologies, Carlsbad, CA, USA). Purified 16S rRNA libraries were pooled and diluted to a concentration of 2 nM. The pool was then denatured with fresh 0.2 M NaOH. Libraries were further diluted according to the manufacturer’s instructions and sequenced on an Illumina MiSeq (Illumina, San Diego, CA, USA) with a 300-cycle V2 nano kit.

### 16S rRNA data analysis

16S rRNA gene sequences were analyzed using Qiime2 software version 2021.2 (44). Sequences were imported as EMPSingleEndSequences and demultiplexed using the demux emp-single command. Sequence quality control was done using DADA2 (45). Default settings were utilized, and the truncation length was set to 250 base pairs. The classify-sklearn (46) command was used to classify the remaining sequences using a pre-trained Naive Bayes classifier trained on Silva 132 99% OTUs (47, 48) from the 515F/806R region. Alpha and beta diversity analyses were also performed using Qiime2 with the core-metrics-phylogenetic command with a sampling depth of 1,000. Chao1 index and Shannon entropy values were calculated using their respective commands as listed on the alpha and beta diversity explanations and commands forum on qiime2.org.

### Shotgun metagenomic sequencing and data analysis

For shotgun metagenomic sequencing, DNA libraries were prepared using the Nextera XT DNA Library Preparation Kit according to the manufacturer’s protocol (Illumina, San Diego, CA, USA). Paired-end sequencing reads (2 × 300 bp) were generated on an Illumina MiSeq with the MiSeq v3 Reagent Kit (600 cycles) (Illumina, San Diego, CA, USA). The authors utilized the US Department of Energy’s KBase (49) platform to perform quality control, assembly, binning, and annotation of MAGs. Software mentioned in the following paragraphs was utilized on KBase.

Paired-end sequencing reads were first uploaded to the KBase platform, then FastQC (v0.11.9) (50) was utilized to assess read quality. Sequences were then trimmed with Trimmomatic (v0.36) (51). The original sequence length was 35–301, after trimming the sequence length was 36–276. Trimmed sequences were then run through FastQC where most of the modules in the software were now labeled as normal, and some slightly abnormal. Initial taxonomic inference of reads was performed by Kaiju (v1.7.3) (52) using the NCBI BLAST nr+euk reference database in “greedy mode” with a minimum match length of 11, minimum match score of 75, and five allowed mismatches.

Contiguous sequences were assembled with metaSPAdes (v3.15.3) (53), MEGA HIT (v1.2.9) (54), and IDBA-UD (v1.1.3) (55). Assembly with metaSPAdes was run with error correction. MEGA HIT was run with both meta-sensitive and meta-large parameters. IDBA-UD was run with default parameters. The assembled contigs were then compared with KBase’s Compare Assembled Contig Distributions (v1.1.2), where the best assembly was then chosen for further analysis.

Contiguous sequences were then binned with MaxBin2 (v2.2.4) (56), MetaBAT2 (v1.7) (57), and CONCOCT (v1.1) (58). All binning software was used at KBase default parameters. Bins were then optimized using DAS Tool (v1.1.2) (59), with blast as the gene identification tool.

The completeness of and quality of these optimized bins or MAGs were then assessed for utilizing CheckM (v1.4.0) (60). MAGs that were >86% complete were then used for further analysis. MAGs that were generated from contig binning were annotated using RASTtk (v1.073) (61) and Prokka (v1.14.5) (62). These MAGs were also annotated off KBase on the Bacterial and Viral Bioinformatics Resource Center (63). Manual curation of selenium-reducing genes was utilized to determine if genes similar to what has been reported in the literature about SeRB were also found in the metagenomes.

Taxonomic assignments for MAGs were examined with GTDB-tk (v1.7.0) (64) and compared with Kaiju outputs from earlier in the processing stream. The average nucleotide identity was also determined with GTDB-tk.

Selenium reduction genes were found via literature search. NCBI BLAST was used to search for known genes in the MAGs. Genes were considered present if *e*-values were less than e^−50^ and bit scores were greater than 50. See the supplemental material for full gene presence/absence chart (Fig. S2).

## Data Availability

Raw sequences, MAGs, and 16S can be found under the NCBI Bioproject number PRJNA1122175. Raw sample accession numbers are SRR30877662, SRR30877663, and SRR30877661. MAG sequences can be found under the genome accession numbers JBIQOF000000000, JBIQOE000000000, and JBIQOG000000000. 16S sequences can be found under the accession numbers SRR29358234, SRR29358233, and SRR29358235.

## SUPPLEMENTAL MATERIAL

Supplemental figures aem.01222-24-s0001.pdf Figures S1 to S5.

ASM does not own the copyrights to Supplemental Material that may be linked to, or accessed through, an article. The authors have granted ASM a non-exclusive, world-wide license to publish the Supplemental Material files. Please contact the corresponding author directly for reuse.

## References

[1] U.S. Energy Information Administration. Use of Coal - U.S. Energy Information Administration (EIA). Available from: https://www.eia.gov/energyexplained/coal/use-of-coal.php. Retrieved 4 Dec 2023.

[2] Gingerich DB, Mauter MS. 2020. Flue gas desulfurization wastewater composition and implications for regulatory and treatment train design. Environ Sci Technol 54:3783–3792. doi:10.1021/acs.est.9b07433 32146805

[3] U.S. EPA. 2020. Supplemental technical development document for revisions to the effluent limitations guidelines and standards for the steam electric power generating point source category, p EPA–821. U.S. Environmental Protection Agency.

[4] Nancharaiah YV, Lens PNL. 2015. Ecology and biotechnology of selenium-respiring bacteria. Microbiol Mol Biol Rev 79:61–80. doi:10.1128/MMBR.00037-14 25631289 PMC4402961

[5] Cheng C-M, Hack P, Chu P, Chang Y-N, Lin T-Y, Ko C-S, Chiang P-H, He C-C, Lai Y-M, Pan W-P. 2009. Partitioning of mercury, arsenic, selenium, boron, and chloride in a full-scale coal combustion process equipped with selective catalytic reduction, electrostatic precipitation, and flue gas desulfurization systems . Energy Fuels 23:4805–4816. doi:10.1021/ef900293u

[6] EPRI. EPRI Technical Manual: Guidance for Assessing Wastewater Impacts of FGD Scrubbers. Available from: https://www.epri.com/research/products/000000000001013313. Retrieved 26 Jun 2023.

[7] Wadhwani SA, Shedbalkar UU, Singh R, Chopade BA. 2016. Biogenic selenium nanoparticles: current status and future prospects. Appl Microbiol Biotechnol 100:2555–2566. doi:10.1007/s00253-016-7300-7 26801915

[8] Wang D, Rensing C, Zheng S. 2022. Microbial reduction and resistance to selenium: mechanisms, applications and prospects. J Hazard Mater 421:126684. doi:10.1016/j.jhazmat.2021.126684 34339989

[9] Bini E, Rauschenbach I, Narasingarao P, Starovoytov V, Hauser L, Jeffries CD, Land M, Bruce D, Detter C, Goodwin L, Han S, Held B, Tapia R, Copeland A, Ivanova N, Mikhailova N, Nolan M, Pati A, Pennacchio L, Pitluck S, Woyke T, Häggblom M. 2011. Complete genome sequence of Desulfurispirillum indicum strain S5^T^. Stand Genomic Sci 5:371–378. doi:10.4056/sigs.2425302 22675586 PMC3368425

[10] Li D-B, Cheng Y-Y, Wu C, Li W-W, Li N, Yang Z-C, Tong Z-H, Yu H-Q. 2014. Selenite reduction by Shewanella oneidensis MR-1 is mediated by fumarate reductase in periplasm. Sci Rep 4:3735. doi:10.1038/srep03735 24435070 PMC3894562

[11] Macy JM, Lawson S, DeMoll-Decker H. 1993. Bioremediation of selenium oxyanions in San Joaquin drainage water using Thauera selenatis in a biological reactor system. Appl Microbiol Biotechnol 40:588–594. doi:10.1007/BF00175752

[12] EPA US. 2015. Technical development document for the effluent limitations guidelines and standards for the steam electric power generating point source category, p EPA–821. U.S. Environmental Protection Agency.

[13] Brown BP, Brown SR, Senko JM. 2012. Microbial communities associated with wet flue gas desulfurization systems. Front Microbiol 3:412. doi:10.3389/fmicb.2012.00412 23226147 PMC3510643

[14] Wells CL, Wilkins TD. 1996. Clostridia: Sporeforming Anaerobic Bacilli. In Baron S (ed), Medical Microbiology, 4th edition. University of Texas Medical Branch at Galveston, Galveston (TX). 21413315

[15] BALCH WE, SCHOBERTH S, TANNER RS, WOLFE RS. 1977. Acetobacterium, a new genus of hydrogen-oxidizing, carbon dioxide-reducing, anaerobic bacteria. Int J Syst Evol Microbiol 27:355–361.

[16] Macy JM, Michel TA, Kirsch DG. 1989. Selenate reduction by a Pseudomonas species: a new mode of anaerobic respiration. FEMS Microbiol Lett 52:195–198. doi:10.1016/0378-1097(89)90195-x 2513248

[17] Subedi G, Taylor J, Hatam I, Baldwin SA. 2017. Simultaneous selenate reduction and denitrification by a consortium of enriched mine site bacteria. Chemosphere 183:536–545. doi:10.1016/j.chemosphere.2017.05.144 28570897

[18] Douglas AE. 2020. The microbial exometabolome: ecological resource and architect of microbial communities. Philos Trans R Soc Lond B Biol Sci 375:20190250. doi:10.1098/rstb.2019.0250 32200747 PMC7133521

[19] D’Souza G, Shitut S, Preussger D, Yousif G, Waschina S, Kost C. 2018. Ecology and evolution of metabolic cross-feeding interactions in bacteria. Nat Prod Rep 35:455–488. doi:10.1039/c8np00009c 29799048

[20] Pinu FR, Granucci N, Daniell J, Han T-L, Carneiro S, Rocha I, Nielsen J, Villas-Boas SG. 2018. Metabolite secretion in microorganisms: the theory of metabolic overflow put to the test. Metabolomics (Los Angel) 14:43. doi:10.1007/s11306-018-1339-7 30830324

[21] Yu JSL, Correia-Melo C, Zorrilla F, Herrera-Dominguez L, Wu MY, Hartl J, Campbell K, Blasche S, Kreidl M, Egger A-S, Messner CB, Demichev V, Freiwald A, Mülleder M, Howell M, Berman J, Patil KR, Alam MT, Ralser M. 2022. Microbial communities form rich extracellular metabolomes that foster metabolic interactions and promote drug tolerance. Nat Microbiol 7:542–555. doi:10.1038/s41564-022-01072-5 35314781 PMC8975748

[22] Schoch CL, Ciufo S, Domrachev M, Hotton CL, Kannan S, Khovanskaya R, Leipe D, Mcveigh R, O’Neill K, Robbertse B, Sharma S, Soussov V, Sullivan JP, Sun L, Turner S, Karsch-Mizrachi I. 2020. Microbial communities form rich extracellular metabolomes that foster metabolic interactions and promote drug tolerance. Nat Microbiol. doi:10.1093/database/baaa062 PMC8975748 35314781

[23] Patel S, Gupta RS. 2020. A phylogenomic and comparative genomic framework for resolving the polyphyly of the genus Bacillus: proposal for six new genera of Bacillus species, Peribacillus gen. nov., Cytobacillus gen. nov., Mesobacillus gen. nov., Neobacillus gen. nov., Metabacillus gen. nov. and Alkalihalobacillus gen. nov. Int J Syst Evol Microbiol 70:406–438. doi:10.1099/ijsem.0.003775 31617837

[24] Yamamura S, Yamashita M, Fujimoto N, Kuroda M, Kashiwa M, Sei K, Fujita M, Ike M. 2007. Bacillus selenatarsenatis sp. nov., a selenate- and arsenate-reducing bacterium isolated from the effluent drain of a glass-manufacturing plant. Int J Syst Evol Microbiol 57:1060–1064. doi:10.1099/ijs.0.64667-0 17473259

[25] Kashiwa M, Ike M, Mihara H, Esaki N, Fujita M. 2001. Removal of soluble selenium by a selenate-reducing bacterium Bacillus sp. SF-1. Biofactors 14:261–265. doi:10.1002/biof.5520140132 11568463

[26] Ogg CD, Patel BKC. 2009. Thermotalea metallivorans gen. nov., sp. nov., a thermophilic, anaerobic bacterium from the Great Artesian Basin of Australia aquifer. Int J Syst Evol Microbiol 59:964–971. doi:10.1099/ijs.0.004218-0 19406776

[27] Hong H, Kim S-J, Min U-G, Lee Y-J, Kim S-G, Roh SW, Kim J-G, Na J-G, Rhee S-K. 2015. Anaerosolibacter carboniphilus gen. nov., sp. nov., a strictly anaerobic iron-reducing bacterium isolated from coal-contaminated soil. Int J Syst Evol Microbiol 65:1480–1485. doi:10.1099/ijs.0.000124 25701849

[28] Dong Y, Sanford RA, Boyanov MI, Kemner KM, Flynn TM, O’Loughlin EJ, Locke RA, Weber JR, Egan SM, Fouke BW. 2016. Tepidibacillus decaturensis sp. nov., a microaerophilic, moderately thermophilic iron-reducing bacterium isolated from 1.7 km depth groundwater. Int J Syst Evol Microbiol 66:3964–3971. doi:10.1099/ijsem.0.001295 27406851

[29] Podosokorskaya OA, Merkel AY, Gavrilov SN, Fedoseev I, Heerden E van, Cason ED, Novikov AA, Kolganova TV, Korzhenkov AA, Bonch-Osmolovskaya EA, Kublanov IV. 2016. Tepidibacillus infernus sp. nov., a moderately thermophilic, selenate- and arsenate-respiring hydrolytic bacterium isolated from a gold mine, and emended description of the genus Tepidibacillus. Int J Syst Evol Microbiol 66:3189–3194. doi:10.1099/ijsem.0.001166 27216447

[30] Gingerich DB, Grol E, Mauter MS. 2018. Fundamental challenges and engineering opportunities in flue gas desulfurization wastewater treatment at coal fired power plants. Environ Sci: Water Res Technol 4:909–925. doi:10.1039/C8EW00264A

[31] Krafft T, Bowen A, Theis F, Macy JM. 2000. Cloning and sequencing of the genes encoding the periplasmic-cytochrome B -containing selenate reductase of Thauera selenatis . DNA Seq 10:365–377. doi:10.3109/10425170009015604 10826693

[32] Kuroda M, Yamashita M, Miwa E, Imao K, Fujimoto N, Ono H, Nagano K, Sei K, Ike M. 2011. Molecular cloning and characterization of the srdBCA operon, encoding the respiratory selenate reductase complex, from the selenate-reducing bacterium Bacillus selenatarsenatis SF-1. J Bacteriol 193:2141–2148. doi:10.1128/JB.01197-10 21357486 PMC3133095

[33] Wells M, McGarry J, Gaye MM, Basu P, Oremland RS, Stolz JF. 2019. Respiratory selenite reductase from Bacillus selenitireducens strain MLS10. J Bacteriol 201:00614–00618. doi:10.1128/JB.00614-18 PMC6416917 30642986

[34] Oremland RS, Herbel MJ, Blum JS, Langley S, Beveridge TJ, Ajayan PM, Sutto T, Ellis AV, Curran S. 2004. Structural and spectral features of selenium nanospheres produced by Se-respiring bacteria. Appl Environ Microbiol 70:52–60. doi:10.1128/AEM.70.1.52-60.2004 14711625 PMC321302

[35] Sochacki KA, Shkel IA, Record MT, Weisshaar JC. 2011. Protein diffusion in the periplasm of E. coli under osmotic stress. Biophys J 100:22–31. doi:10.1016/j.bpj.2010.11.044 21190653 PMC3010016

[36] Sun J, Rutherford ST, Silhavy TJ, Huang KC. 2022. Physical properties of the bacterial outer membrane. Nat Rev Microbiol 20:236–248. doi:10.1038/s41579-021-00638-0 34732874 PMC8934262

[37] The Gram-negative bacterial periplasm: Size matters - PMC. 2024. Available from: https://www.ncbi.nlm.nih.gov/pmc/articles/PMC5771553/. Retrieved 16 Apr 2024. 10.1371/journal.pbio.2004935 PMC5771553 29342145

[38] Abràmoff DMD. 2004. Image Processing with ImageJ. Biophotonics Int 11:36–42.

[39] Pace HE, Rogers NJ, Jarolimek C, Coleman VA, Higgins CP, Ranville JF. 2011. Determining transport efficiency for the purpose of counting and sizing nanoparticles via single particle inductively coupled plasma mass spectrometry. Anal Chem 83:9361–9369. doi:10.1021/ac201952t 22074486 PMC3410750

[40] Bland GD, Battifarano M, Liu Q, Yang X, Lu D, Jiang G, Lowry GV. 2023. Single-particle metal fingerprint analysis and machine learning pipeline for source apportionment of metal-containing fine particles in air. Environ Sci Technol Lett 10:1023–1029. doi:10.1021/acs.estlett.2c00835

[41] Loosli F, Wang J, Rothenberg S, Bizimis M, Winkler C, Borovinskaya O, Flamigni L, Baalousha M. 2019. Sewage spills are a major source of titanium dioxide engineered (nano)-particles into the environment. Environ Sci Nano 6:763–777. doi:10.1039/C8EN01376D 31853367 PMC6919659

[42] Maeda H, Fujimoto C, Haruki Y, Maeda T, Kokeguchi S, Petelin M, Arai H, Tanimoto I, Nishimura F, Takashiba S. 2003. Quantitative real-time PCR using TaqMan and SYBR Green for Actinobacillus actinomycetemcomitans, Porphyromonas gingivalis, Prevotella intermedia, tetQ gene and total bacteria. FEMS Immunol Med Microbiol 39:81–86. doi:10.1016/S0928-8244(03)00224-4 14557000

[43] Caporaso JG, Lauber CL, Walters WA, Berg-Lyons D, Lozupone CA, Turnbaugh PJ, Fierer N, Knight R. 2011. Global patterns of 16S rRNA diversity at a depth of millions of sequences per sample. Proc Natl Acad Sci USA 108 Suppl 1:4516–4522. doi:10.1073/pnas.1000080107 20534432 PMC3063599

[44] Bolyen E, Rideout JR, Dillon MR, Bokulich NA, Abnet CC, Al-Ghalith GA, Alexander H, Alm EJ, Arumugam M, Asnicar F, et al.. 2019. Reproducible, interactive, scalable and extensible microbiome data science using QIIME 2. Nat Biotechnol 37:852–857. doi:10.1038/s41587-019-0209-9 31341288 PMC7015180

[45] Callahan BJ, McMurdie PJ, Rosen MJ, Han AW, Johnson AJA, Holmes SP. 2016. DADA2: high-resolution sample inference from Illumina amplicon data. Nat Methods 13:581–583. doi:10.1038/nmeth.3869 27214047 PMC4927377

[46] Pedregosa F, Varoquaux G, Gramfort A. 2011. Scikit-learn: Machine Learning in Python. J. Machine Learn. Res 12:2825–2830.

[47] Quast C, Pruesse E, Yilmaz P, Gerken J, Schweer T, Yarza P, Peplies J, Glöckner FO. 2013. The SILVA ribosomal RNA gene database project: improved data processing and web-based tools. Nucleic Acids Res 41:D590–D596. doi:10.1093/nar/gks1219 23193283 PMC3531112

[48] Yilmaz P, Parfrey LW, Yarza P, Gerken J, Pruesse E, Quast C, Schweer T, Peplies J, Ludwig W, Glöckner FO. 2014. The SILVA and “all-species living tree project (LTP)” taxonomic frameworks. Nucl Acids Res 42:D643–D648. doi:10.1093/nar/gkt1209 24293649 PMC3965112

[49] Arkin AP, Cottingham RW, Henry CS, Harris NL, Stevens RL, Maslov S, Dehal P, Ware D, Perez F, Canon S, et al.. 2018. KBase: the United States department of energy systems biology knowledgebase. Nat Biotechnol 36:566–569. doi:10.1038/nbt.4163 29979655 PMC6870991

[50] 2023. Babraham bioinformatics - FastQC a quality control tool for high throughput sequence data. Available from: https://www.bioinformatics.babraham.ac.uk/projects/fastqc. Retrieved 16 Feb 2023.

[51] Bolger AM, Lohse M, Usadel B. 2014. Trimmomatic: a flexible trimmer for Illumina sequence data. Bioinformatics 30:2114–2120. doi:10.1093/bioinformatics/btu170 24695404 PMC4103590

[52] Menzel P, Ng KL, Krogh A. 2016. Fast and sensitive taxonomic classification for metagenomics with Kaiju. Nat Commun 7:11257. doi:10.1038/ncomms11257 27071849 PMC4833860

[53] Nurk S, Meleshko D, Korobeynikov A, Pevzner PA. 2017. metaSPAdes: a new versatile metagenomic assembler. Genome Res 27:824–834. doi:10.1101/gr.213959.116 28298430 PMC5411777

[54] Li D, Liu C-M, Luo R, Sadakane K, Lam T-W. 2015. MEGAHIT: an ultra-fast single-node solution for large and complex metagenomics assembly via succinct de Bruijn graph . Bioinformatics 31:1674–1676. doi:10.1093/bioinformatics/btv033 25609793

[55] Peng Y, Leung HCM, Yiu SM, Chin FYL. 2012. IDBA-UD: a de novo assembler for single-cell and metagenomic sequencing data with highly uneven depth. Bioinformatics 28:1420–1428. doi:10.1093/bioinformatics/bts174 22495754

[56] Wu Y-W, Simmons BA, Singer SW. 2016. MaxBin 2.0: an automated binning algorithm to recover genomes from multiple metagenomic datasets. Bioinformatics 32:605–607. doi:10.1093/bioinformatics/btv638 26515820

[57] Kang DD, Li F, Kirton E, Thomas A, Egan R, An H, Wang Z. 2019. MetaBAT 2: an adaptive binning algorithm for robust and efficient genome reconstruction from metagenome assemblies. PeerJ 7:e7359. doi:10.7717/peerj.7359 31388474 PMC6662567

[58] Alneberg J, Bjarnason BS, de Bruijn I, Schirmer M, Quick J, Ijaz UZ, Lahti L, Loman NJ, Andersson AF, Quince C. 2014. Binning metagenomic contigs by coverage and composition. Nat Methods 11:1144–1146. doi:10.1038/nmeth.3103 25218180

[59] Sieber CMK, Probst AJ, Sharrar A, Thomas BC, Hess M, Tringe SG, Banfield JF. 2018. Recovery of genomes from metagenomes via a dereplication, aggregation and scoring strategy. Nat Microbiol 3:836–843. doi:10.1038/s41564-018-0171-1 29807988 PMC6786971

[60] Parks DH, Imelfort M, Skennerton CT, Hugenholtz P, Tyson GW. 2015. CheckM: assessing the quality of microbial genomes recovered from isolates, single cells, and metagenomes. Genome Res 25:1043–1055. doi:10.1101/gr.186072.114 25977477 PMC4484387

[61] Brettin T, Davis JJ, Disz T, Edwards RA, Gerdes S, Olsen GJ, Olson R, Overbeek R, Parrello B, Pusch GD, Shukla M, Thomason JA, Stevens R, Vonstein V, Wattam AR, Xia F. 2015. RASTtk: a modular and extensible implementation of the RAST algorithm for building custom annotation pipelines and annotating batches of genomes. Sci Rep 5:1. doi:10.1038/srep08365 PMC4322359 25666585

[62] Seemann T. 2014. Prokka: rapid prokaryotic genome annotation. Bioinformatics 30:2068–2069. doi:10.1093/bioinformatics/btu153 24642063

[63] Olson RD, Assaf R, Brettin T, Conrad N, Cucinell C, Davis JJ, Dempsey DM, Dickerman A, Dietrich EM, Kenyon RW, et al.. 2023. Introducing the bacterial and viral bioinformatics resource center (BV-BRC): a resource combining PATRIC, IRD and ViPR. Nucleic Acids Res 51:D678–D689. doi:10.1093/nar/gkac1003 36350631 PMC9825582

[64] Chaumeil P-A, Mussig AJ, Hugenholtz P, Parks DH. 2020. GTDB-Tk: a toolkit to classify genomes with the genome taxonomy database. Bioinformatics 36:1925–1927. doi:10.1093/bioinformatics/btz848 PMC7703759 31730192

