# Supplementary material for: Novel anaerobic selenium oxyanion reducers native to FGD wastewater for enhanced selenium removal
Source: Appl Environ Microbiol. 2025 Mar 14;91(4):e01222-24. doi: 10.1128/aem.01222-24 (PMC12016495; doi:10.1128/aem.01222-24)

Supplementary
S1:

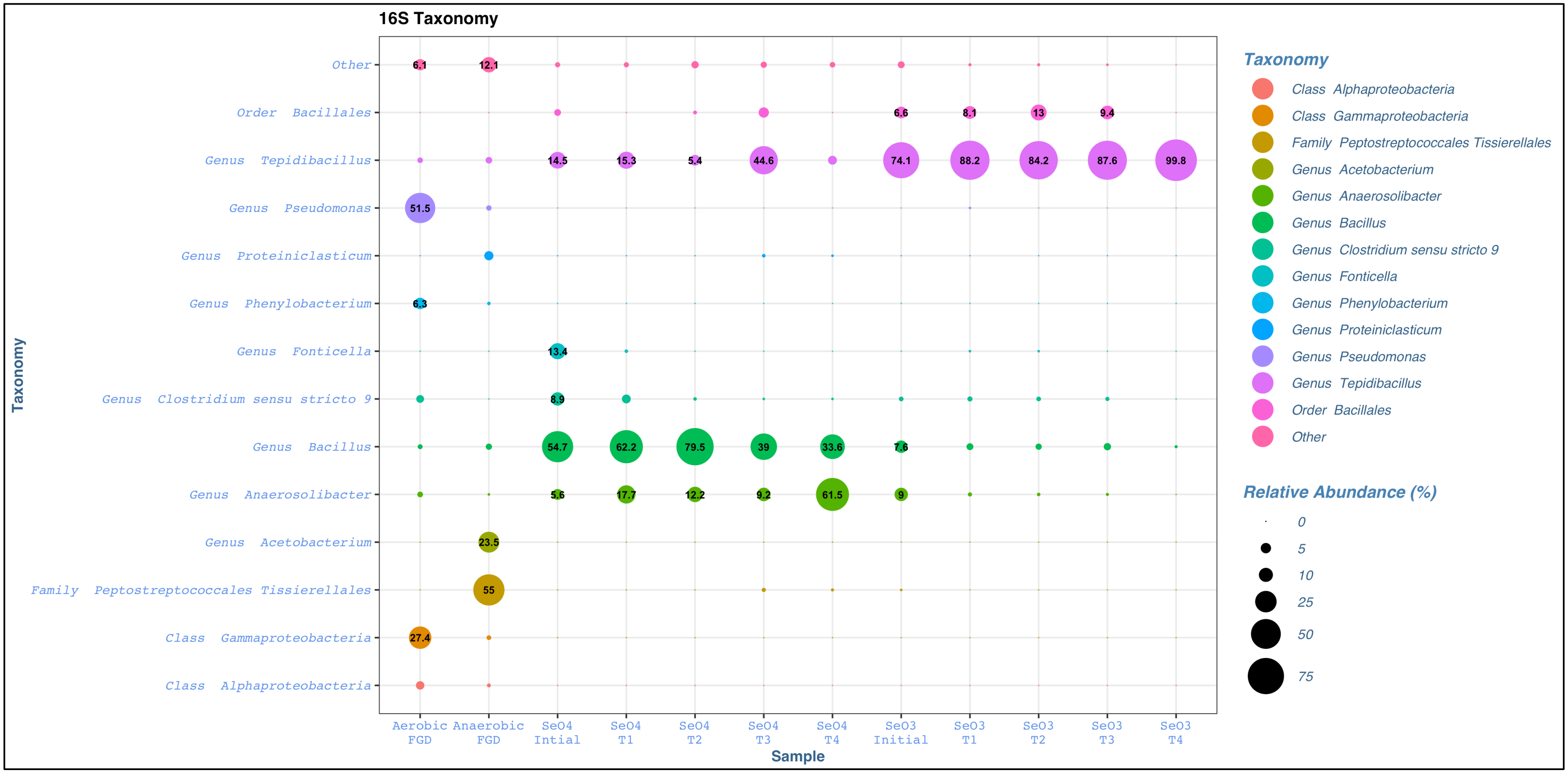

Figure S1: 16S Taxonomy and Relative Abundance of Samples
Microbial Relative abundances of all samples. Each sample was done in triplicate. Data represents normalized data. The larger the bubble the more relatively abundant the organism. Other represents all organisms that make less than 2% of the relative abundance of the sample that have not been mentioned on the Y-axis previously.

S2: Full Presence/Absence Selenium Genes in Recovered MAGs
Presence is indicated by black shading for relevant gene.

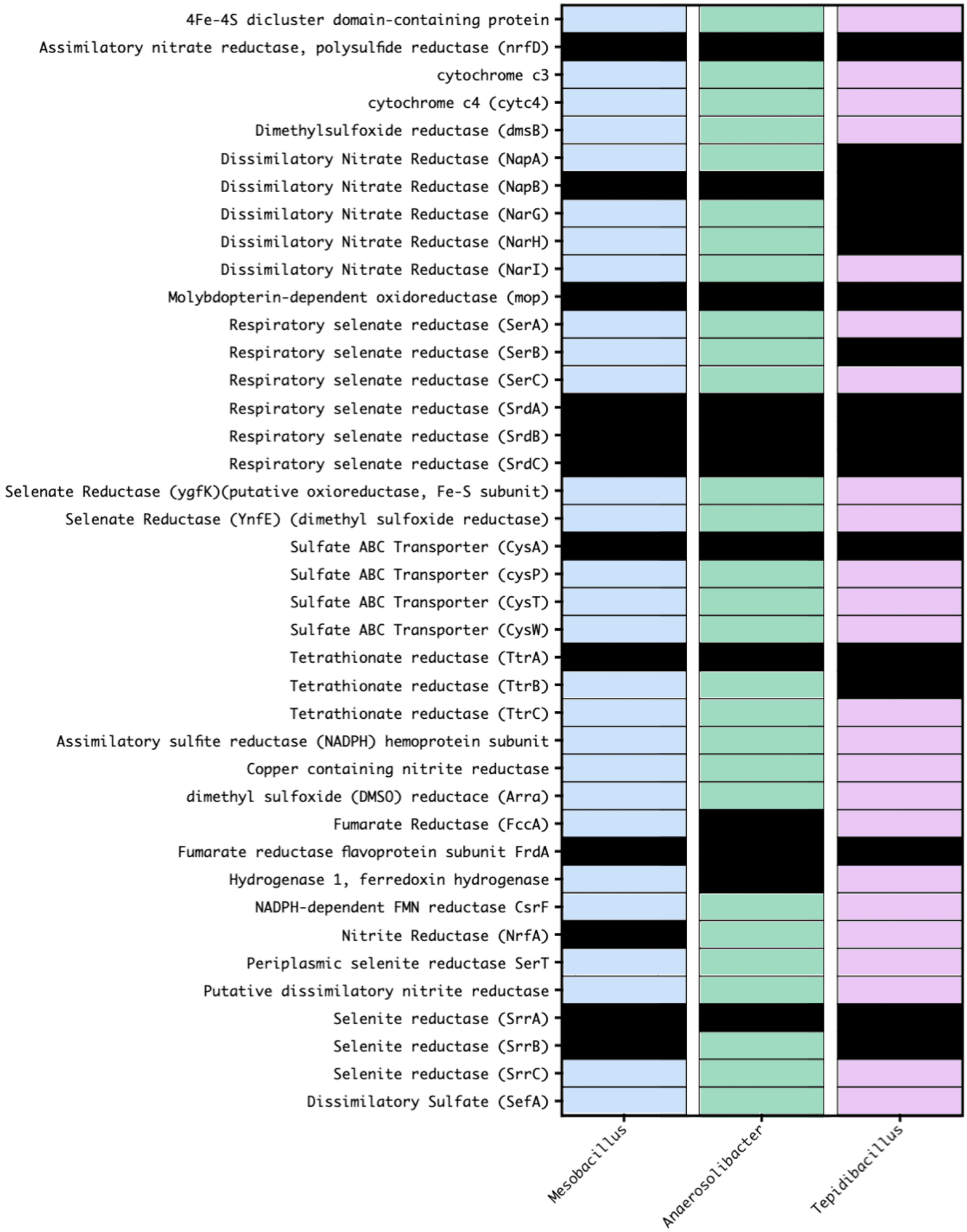

S3:SEM-EDS Image showing selenium.
Silicon peak is shown due to interference from glass cover slip used to mount samples.

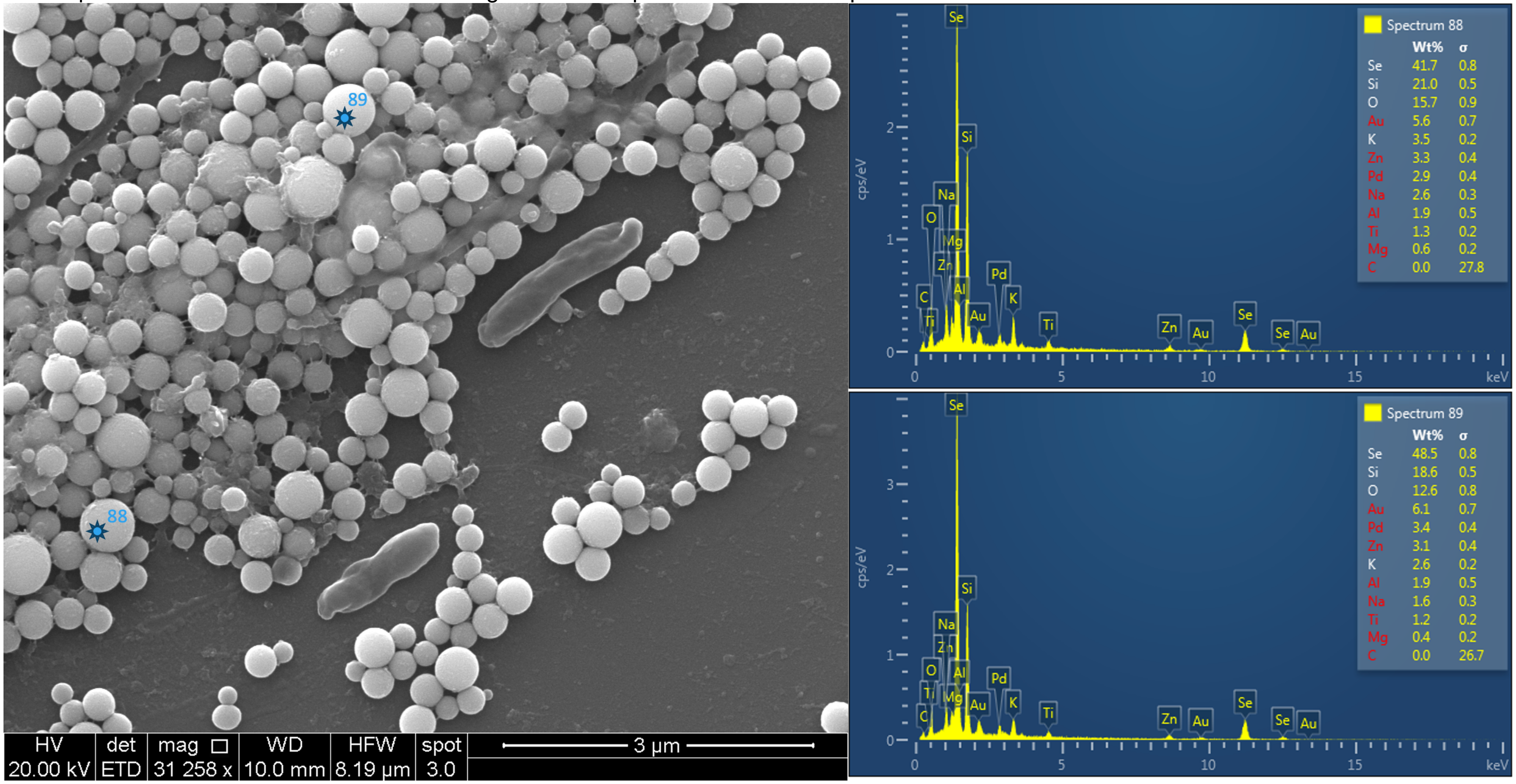

S4:XRD analysis of selenium sample from Selenite TR3 reactor depicting hexagonal selenium.

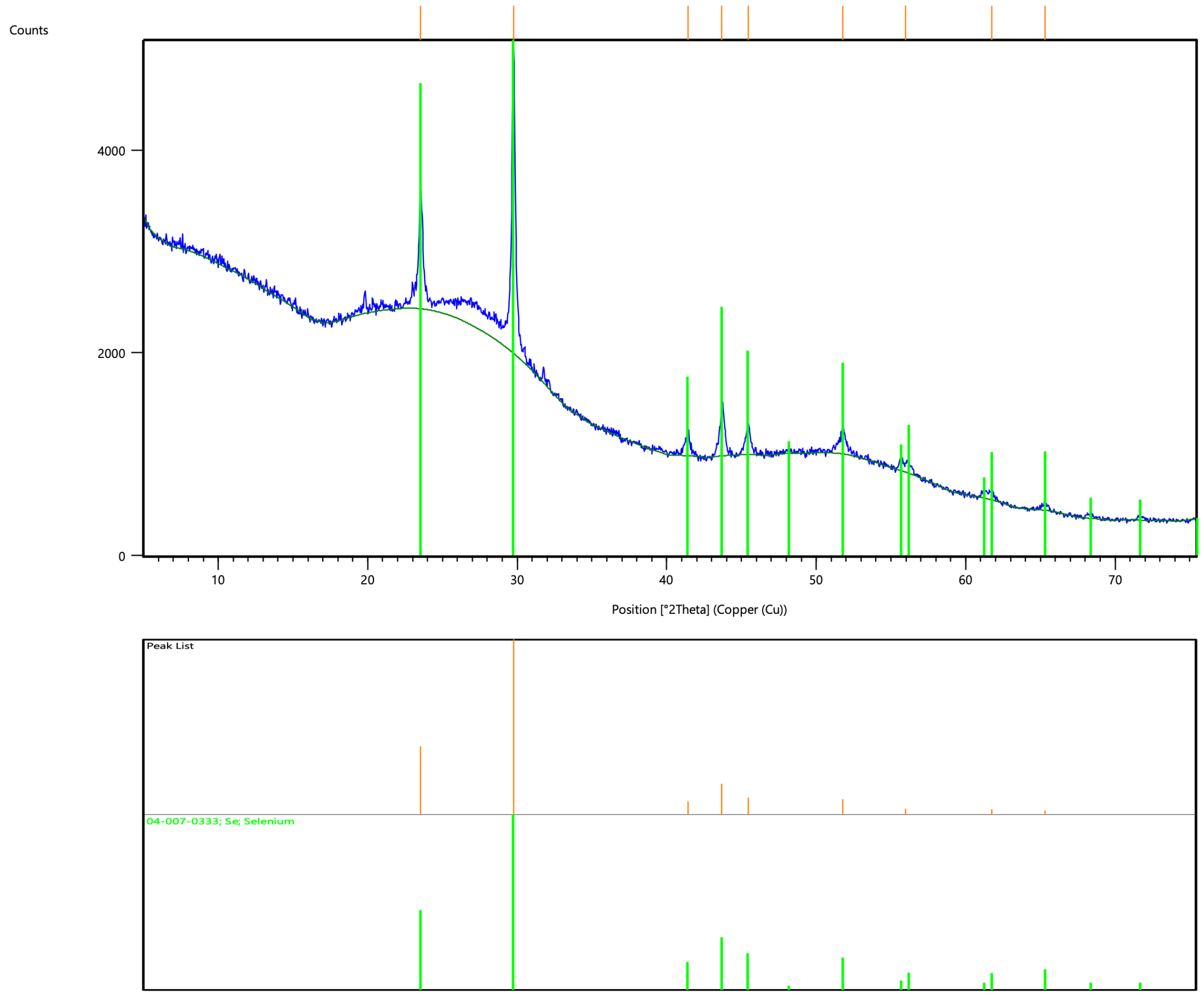

S5: spICP-TOFMS and SEM Particle Analysis:
The number of particles next to the transfer is the number of particles detected by the TOF. SEM data is for 500particles in all samples.

Selenate

TR0 – 2832 particles

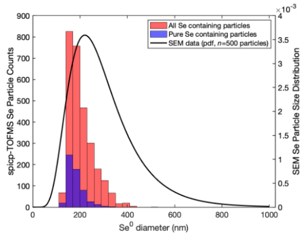

TR1 – 19434 particles

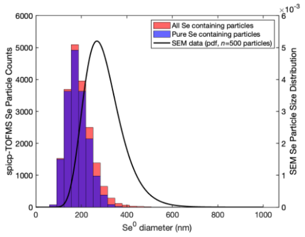

TR2 – 7373 particles

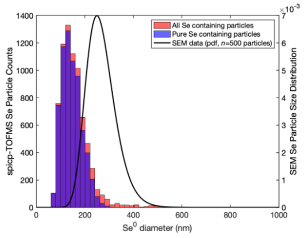

TR3 – 8473 particles

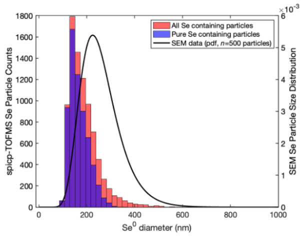

TR4 – 7183 particles

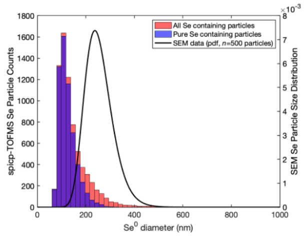

Selenite

TR0 – 19521 particles

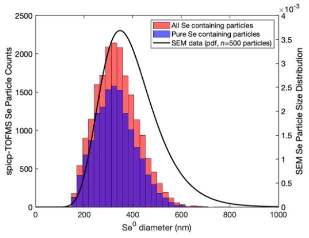

TR1 – 11298 particles

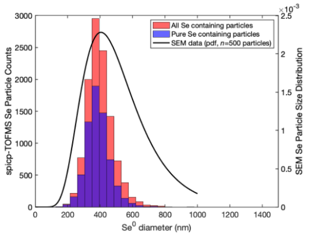

TR2 – 9536 particles

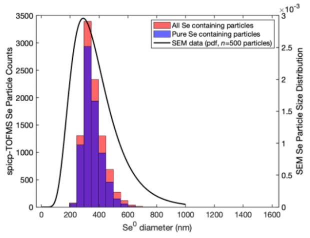

TR3 – 8228 particles

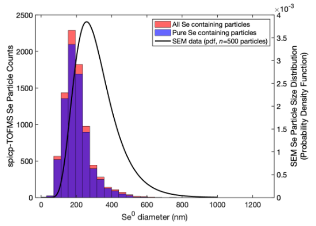

TR4 – 8069 particles

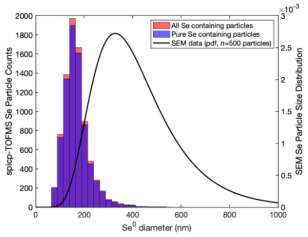

Supplement: Supplemental figures — Figures S1 to S5. [file aem.01222-24-s0001.pdf]
